# LRG1 Drives Pathological Angiogenesis by Disrupting Neutrophil Mitochondrial Homeostasis in Bladder Cancer

**DOI:** 10.1002/advs.76604

**Published:** 2026-07-13

**Authors:** Dongshan Chen, Cong Zhang, Song Xue, Yuan Zeng, Haochen Cui, Chenfei Wang, Jiayi Feng, Lei Yan, Yuanwei Zang

**Affiliations:** ^1^ Department of Urology Qilu Hospital of Shandong University Jinan China; ^2^ Shandong Academy of Pharmaceutical Sciences Jinan China; ^3^ College of Foreign Languages Shandong University of Traditional Chinese Medicine Jinan China; ^4^ Biomedical Sciences College Shandong First Medical University Jinan China

**Keywords:** angiogenesis, bladder cancer, LRG1, mitochondrial homeostasis, neutrophil

## Abstract

Abnormal tumor vasculature creates a permissive microenvironment that fuels the malignant progression of bladder cancer (BCa). While Leucine‐rich alpha‐2 glycoprotein 1 (LRG1) is known to regulate angiogenesis, its specific role in remodeling the BCa microenvironment remains poorly defined.We integrated single‐cell RNA sequencing (scRNA‐seq) with bulk transcriptomic datasets to identify key cellular subclusters. Functional validation was performed using subcutaneous and orthotopic BCa mouse models, neutrophil depletion, DNase I treatment, and clinical specimens. The molecular interactome was mapped via pull‐down assays, mass spectrometry, and confocal imaging.LRG1 is significantly upregulated in BCa and correlates with hematogenous metastasis and poor prognosis. Mechanistically, tumor‐derived LRG1 binds to Annexin A2 (ANXA2) on neutrophils through its LRR domain, impeding the mitochondrial translocation of Akt and triggering mtROS‐dependent release of Neutrophil Extracellular Traps (NETs). These NETs directly cause vascular destabilization by stripping mural cell coverage. Blockade of the LRG1‐NETosis axis induces vascular normalization, effectively overcoming microenvironmental barriers to increase drug delivery and T‐cell infiltration, thereby profoundly sensitizing BCa to cisplatin and anti‐PD‐1 therapy.The LRG1‐neutrophil‐NETosis axis is a critical driver of vascular dysfunction and therapeutic resistance in BCa. Targeting this axis represents a promising translational strategy to improve clinical outcomes.

## Graphical abstract

In bladder cancer, LRG1 binds to ANXA2 to trigger mitochondrial ROS‐dependent NETosis. This pathogenic cascade actively uncouples endothelial‐mural cell interactions, driving profound vascular destabilization. Consequently, targeting the LRG1‐ANXA2 axis attenuates the neutrophil burden and induces structural vascular normalization, offering a powerful strategy to enhance systemic chemo‐immunotherapy efficacy.

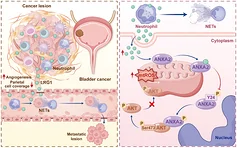

**Abbreviations**
ANXA2: Annexin A2
BCa: bladder cancer
cfDNA: cell‐free DNA
CM: conditioned media
CNV: copy number variation
Co‐IP: co‐immunoprecipitation
ELISA: enzyme‐linked immunosorbent assay
GEO: Gene Expression Omnibus
GSVA: Gene Set Variation Analysis
H&E: hematoxylin and eosin
H3Cit: citrullinated histone H3
HM: hematogenous metastasis
HUVEC: human umbilical vein endothelial cells
IACUC: Institutional Animal Care and Use Committee
IHC: immunohistochemistry
LPS: lipopolysaccharide
LRG1: leucine‐rich alpha‐2 glycoprotein 1
MPO: myeloperoxidase
mtROS: mitochondrial reactive oxygen species
NE: neutrophil elastase
NETs: neutrophil extracellular traps
NMF: non‐negative matrix factorization
OE: overexpression
PD‐1: programmed cell death protein 1
PMA: phorbol 12‐myristate 13‐acetate
qRT‐PCR: quantitative real‐time polymerase chain reaction
rhLRG1: recombinant human LRG1
scRNA‐seq: single‐cell RNA sequencing
STR: short tandem repeat
TCGA: The Cancer Genome Atlas
UMAP: uniform manifold approximation and projection

## 1 Background

The clinical management of advanced bladder cancer faces a persistent bottleneck where the abundant tumor vasculature remains functionally chaotic and poorly perfused [1]. This paradox creates a hypoxic and immunosuppressive microenvironment that not only impedes the effective delivery of cytotoxic agents like cisplatin but also blunts the efficacy of immune checkpoint inhibitors by restricting T‐cell infiltration [2, 3]. Although anti‐angiogenic therapies targeting the VEGF pathway have been explored to rectify these structural aberrations through vascular normalization, their clinical success in bladder cancer remains limited due to transient responses and compensatory resistance mechanisms [4]. Therefore, deciphering the non‐canonical drivers of pathological angiogenesis that sustain this vascular dysregulation is imperative for developing durable combination therapies.

Leucine‐rich alpha‐2‐glycoprotein (LRG1) has recently emerged as a distinct vascular remodeling factor that complements the classical VEGF axis [5]. Unlike physiological angiogenic mediators, LRG1 is specifically induced in pathogenic contexts where it destabilizes endothelial junctions and promotes vessel leakage to facilitate metastatic dissemination [6, 7]. While LRG1 is known to modulate TGF‐β signaling in endothelial cells, its broader role in orchestrating the multicellular ecology of the tumor microenvironment remains underappreciated [8]. To deconstruct the cellular heterogeneity driving this phenotype, we employed single‐cell RNA sequencing to map the immune‐vascular landscape of bladder cancer. This high‐resolution analysis unveiled a striking and specific enrichment of neutrophils and neutrophil extracellular traps (NETs) signatures within LRG1‐high microenvironments, identifying innate immune activation as a potential downstream effector of LRG1 signaling.

Tumor‐associated neutrophils are increasingly implicated in the remodeling of the tumor microenvironment, particularly through the release of NETs. While accumulating evidence indicates that NETs may support angiogenesis, the precise mechanisms by which they subvert vascular structural integrity remain incompletely defined [9, 10]. Furthermore, a central unresolved question is how NETosis is triggered within the sterile, metabolic‐stress environment of the tumor. Emerging evidence points to the loss of mitochondrial homeostasis and a subsequent mitochondrial ROS (mtROS) burst as a prerequisite for this “suicidal” activation [11]. Yet, the upstream extracellular cues that initiate this metabolic reprogramming in neutrophils remain unidentified. Here, we investigated whether LRG1 functions as a critical metabolic disruptor that hijacks neutrophil mitochondrial function to drive this pathological cascade.

In this study, we delineate a previously unrecognized paracrine‐immune axis driving vascular dysregulation in bladder cancer. We demonstrate that secreted LRG1 orchestrates a pro‐angiogenic yet destabilizing microenvironment by promoting neutrophil recruitment and triggering mtROS‐dependent NETosis. These LRG1‐elicited NETs function as key effectors that drive robust neovascularization while concurrently compromising vascular structural integrity by reducing mural cell coverage.

## 2 Materials and Methods

### 2.1 Bioinformatics Analysis

For single‐cell RNA sequencing (scRNA‐seq) analysis, four public bladder cancer datasets (GSE301651, GSE267718, GSE225190, and GSE175526) were integrated. Raw data processing via the Seurat package involved filtering low‐quality cells based on transcript/gene counts and mitochondrial fraction, followed by ambient RNA removal using decontX. Batch effects were mitigated utilizing harmony prior to unsupervised clustering and cell lineage annotation (Figure S1A). Malignant cells were distinguished from normal epithelium through copy number variation (CNV) profiling via inferCNV, utilizing immune and stromal cells as diploid references for k‐means clustering. To parse malignant heterogeneity, Non‐negative Matrix Factorization (NMF) was performed using GeneNMF to identify distinct tumor meta‐programs and subclusters. Crucially, the top 100 marker genes derived from these scRNA‐seq subclusters were subsequently applied to bulk RNA‐seq datasets (TCGA, GSE188715, and GSE13507) to evaluate their prognostic impact on patient survival. Concurrently, neutrophils were extracted, reclustered, and assessed for Neutrophil Extracellular Traps (NETs) pathway enrichment using UCell. Finally, Gene Set Variation Analysis (GSVA) was employed on the bulk RNA‐seq data to deconvolute the relative infiltration abundance of the identified subclusters, and Spearman correlation coefficients (stats package) were calculated to delineate the inter‐cellular crosstalk among specific tumor meta‐programs, NETs‐associated neutrophils, and endothelial cells.

### 2.2 Patients and Animals

Clinical specimens, including surgically resected tumor tissues and matched peripheral blood, were retrospectively collected from 35 bladder cancer patients at Qilu Hospital of Shandong University. Peripheral blood from 16 healthy individuals was utilized for clinical comparisons and primary neutrophil isolation. All participants provided written informed consent, and the study was approved by the Institutional Review Board (IRB) of Qilu Hospital in accordance with the Declaration of Helsinki.

For in vivo studies, C57BL/6 mice were acquired from Beijing Vital River Laboratory Animal Technology (Beijing, China). Lrg1‐deficient (Lrg1^−/−^) mice were obtained from Shanghai Model Organisms Center (Shanghai, China). All mice were maintained in specific pathogen‐free facilities. Experimental procedures complied with the NIH Guide for the Care and Use of Laboratory Animals and received approval from the Laboratory Animal Ethical Committee of Qilu Hospital of Shandong University.

### 2.3 Cell Lines and Culture

Human uroepithelial cells (sv‐HUC‐1) and the bladder cancer cell line UMUC3 were obtained from Procell Life Science & Technology (Wuhan, China) in 2024. Other bladder cancer cell lines (T24, J82, 5637, and MB49) and the human leukemia cell line HL‐60 were purchased from Zishan Biotechnology (Shanghai, China) in 2024.

Cells were maintained in their respective media: Ham's F‐12K for sv‐HUC‐1, MEM for J82, RPMI‐1640 for UMUC3, T24, 5637 and MB49, and IMDM for HL‐60. All media were supplemented with 10% fetal bovine serum (FBS) and 1% penicillin‐streptomycin. Cultures were kept at 37°C in a humidified atmosphere containing 5% CO2. All cell lines were authenticated by Short Tandem Repeat (STR) profiling and were confirmed to be negative for mycoplasma contamination before use in experiments.

### 2.4 Lentiviral Infection and Plasmid Transfection

Lentiviral vectors for LRG1 knockdown (sh‐LRG1) and overexpression (lv‐LRG1), along with their corresponding negative controls, were constructed by and purchased from Tsingke Biotechnology (Beijing, China). BCa cells were infected with the lentiviruses in the presence of polybrene, followed by selection with puromycin to establish stable cell lines.

For mechanistic studies, expression plasmids encoding Flag‐tagged full‐length LRG1, LRG1 truncation mutants, and HA‐tagged ANXA2 (including site‐directed mutants) were obtained from the MiaoLing Plasmid Sharing Platform (Wuhan, China). Transient transfections were performed using the jetPRIME transfection reagent (101000046, Polyplus‐transfection, France) according to the manufacturer's instructions.

### 2.5 Immunohistochemistry (IHC)

Paraffin‐embedded tissue sections (4 µm) were deparaffinized, rehydrated, and subjected to heat‐induced antigen retrieval. Following the inhibition of endogenous peroxidase activity and blocking of non‐specific binding, sections were probed overnight at 4°C with primary antibodies against LRG1 (1:200; 13224‐1‐AP, Proteintech) and CD66b (1:200; A22753, ABclonal). Subsequently, sections were incubated with horseradish peroxidase (HRP)‐conjugated secondary antibodies, visualized using a 3,3'‐diaminobenzidine (DAB) substrate kit, and counterstained with hematoxylin. Images were captured using a light microscope.

### 2.6 Western Blotting (WB)

Total protein was extracted from tissues or cells using RIPA lysis buffer supplemented with protease and phosphatase inhibitors. Protein concentration was determined via the BCA assay. Equal amounts of protein lysates were resolved by SDS‐PAGE and transferred onto PVDF membranes. After blocking with 5% non‐fat milk, membranes were probed overnight at 4°C with specific primary antibodies. Following incubation with HRP‐conjugated secondary antibodies, immunoreactive bands were visualized using an enhanced chemiluminescence (ECL) detection system. Detailed information regarding the antibodies used is provided in Table S1.

### 2.7 Immunofluorescence and TUNEL Assay

Immunofluorescence was performed on formalin‐fixed paraffin‐embedded (FFPE) tissue sections and neutrophil smears. Tissue sections underwent deparaffinization, rehydration, heat‐induced antigen retrieval, permeabilization with 0.5% Triton X‐100, and blocking of non‐specific binding. Neutrophil smears were fixed with 4% paraformaldehyde and similarly permeabilized with 0.5% Triton X‐100. To simultaneously visualize LRG1, CD31, and α‐SMA, a multiplex immunofluorescence assay was performed using a Tyramide Signal Amplification (TSA) kit (G1236, Servicebio, China). Subsequently, all samples were incubated overnight at 4°C with specific primary antibodies (Table S2), followed by fluorophore‐conjugated secondary antibodies. Vascular architecture was imaged using a Nexcope NCF9300 confocal microscope, while subcellular co‐localization was analyzed using an Olympus SpinSR10 spinning disk confocal system. Vascular density was quantified by counting CD31‐positive vessels per field, and pericyte coverage was calculated as the ratio of the α‐SMA‐positive area to the total CD31‐positive vascular area using ImageJ software.

Apoptosis in tissue sections was assessed using the In Situ Cell Death Detection Kit, Fluorescein (12156792910, Roche, Mannheim, Germany) according to the manufacturer's instructions.

### 2.8 Electron Microscopy

Scanning electron microscopy (SEM). Fresh mouse tumor tissues were fixed in 2.5% glutaraldehyde, dehydrated through a graded ethanol series, and critical‐point dried. Following gold sputter‐coating, the tumor vasculature was examined using a SU8100 scanning electron microscope (Hitachi, Japan). Particular attention was paid to the presence of intraluminal membranous inclusions and endothelial surface ultrastructure.

Transmission electron microscopy (TEM). Isolated neutrophils were fixed, embedded in epoxy resin, and sectioned (70 nm). Ultrathin sections were stained with uranyl acetate and lead citrate. Mitochondrial ultrastructure, including cristae architecture and swelling, was visualized using a Hitachi transmission electron microscope (Hitachi, Japan).

### 2.9 Neutrophil Isolation and NETs Induction

Human neutrophils were isolated from the peripheral blood of healthy donors using a Neutrophil Extraction Kit (P9040, Solarbio, China) according to the manufacturer's instructions. The purity of isolated neutrophils was verified to be >95% via Wright‐Giemsa staining and flow cytometry (CD16^+^CD66b^+^). To induce NETosis, neutrophils were stimulated with 100 nM Phorbol 12‐myristate 13‐acetate (PMA; RPM0015, ABclonal, China) or recombinant human LRG1 (rh‐LRG1; RP01986, ABclonal, China) for 4 h. NET formation was visualized and quantified using SYTOX Green nucleic acid stain or confirmed by immunofluorescence for MPO and H3Cit. For NET disruption, cells were treated with DNase I (D8070‐15KU, Solarbio, China) to degrade extracellular DNA scaffolds.

### 2.10 Transwell Migration Assays

To evaluate the direct impact of NETs on metastatic potential, bladder cancer cells (5 × 10^4^) were suspended in serum‐free medium and seeded into the upper chambers of 8.0‐µm pore inserts. Isolated NETs, with or without DNase1 digestion, were added directly to the upper chamber and co‐incubated with the cancer cells. The lower chamber was filled with medium supplemented with 10% FBS to serve as a chemoattractant. Following a 24‐h incubation at 37°C, non‐migrated cells on the upper surface were removed. Cells that had migrated to the lower surface were fixed with 4% paraformaldehyde, stained with 0.1% crystal violet, and imaged.

Neutrophil chemotaxis was assessed using 3.0‐µm pore Transwell inserts. Freshly isolated neutrophils (5 × 10^5^) were loaded into the upper chamber. The lower chamber was supplied with a 1:1 mixture of RPMI‐1640 and conditioned medium (CM) harvested from LRG1‐modified tumor cells. After a 3‐h incubation at 37°C, the neutrophils that had transmigrated into the lower compartment were collected and directly counted using a hemocytometer.

### 2.11 Tube Formation and Endothelial Permeability Assays

To evaluate the angiogenic potential of HUVECs, 96‐well plates were coated with 50 µL of Matrigel (RPM0004, Abclonal, China) and allowed to polymerize. HUVECs (1.5 × 10^4^ cells/well) were suspended in medium containing isolated NETs or DNase 1‐digested NETs and seeded onto the solidified matrix. After incubation for 8 h at 37°C, the formation of capillary‐like structures was imaged. The total tube length and the number of master junctions were quantified using the Angiogenesis Analyzer plugin in ImageJ.

HUVEC monolayers were established by seeding 2.5 × 10^4^ cells onto the apical surface of Transwell inserts (0.4 µm pore size) and culturing them for 48 h. The confluent monolayers were then challenged with NETs or DNase I‐treated NETs for 24 h. Paracellular flux was determined by adding TRITC‐Dextran (20 µg/mL) to the apical chamber and measuring the fluorescence of the basolateral fraction after 20 min using a Tecan Infinite E plex reader (Tecan, Switzerland).

### 2.12 Co‐Immunoprecipitation (Co‐IP)

Cells were lysed in IP lysis buffer supplemented with protease and phosphatase inhibitors. The supernatants were collected and incubated overnight at 4°C with specific primary antibodies or isotype‐matched control IgG. Protein A/G magnetic beads were subsequently added to capture the immune complexes. After extensive washing to remove non‐specific binders, the bound proteins were eluted by boiling in SDS loading buffer and subjected to immunoblotting analysis. Detailed information regarding the specific antibodies used for immunoprecipitation is provided in Table S1.

### 2.13 Mass Spectrometry Analysis

Co‐IP was performed by incubating cell lysates with anti‐LRG1, anti‐His, or control IgG antibodies, followed by Protein A/G bead capture. Eluted protein complexes were separated by SDS‐PAGE and subjected to in‐gel tryptic digestion. Peptides were resolved via an EASY‐nanoLC 1200 system coupled to an Orbitrap Fusion Lumos mass spectrometer using a 30‐min gradient. Raw spectra were searched against the UniProt human database using PEAKS Studio 10.6. Specific interactors were defined as proteins identified (1% FDR) in the LRG1 or His‐tag pull‐down groups but absent in the IgG control.

### 2.14 Single‐Cell RNA Sequencing (scRNA‐seq) Analysis

Tissue dissociation was performed using the MACS Tumor Dissociation Kit (Miltenyi Biotec), followed by red blood cell lysis. High‐viability single‐cell suspensions (>80%) were loaded onto the DNBelab C4 microfluidic platform (MGI) for droplet generation. scRNA‐seq libraries were constructed using the DNBelab C Series Library Kit according to the manufacturer's protocol, which involved reverse transcription, cDNA amplification, and circularization. Sequencing was conducted on a DNBSEQ‐T7 system utilizing a customized read structure (30 bp for barcode/UMI and 100 bp for cDNA). Raw reads were processed using the DNBelab C Series pipeline, where alignment to the GRCh38 reference was performed via STAR. Valid cells were distinguished from background noise using the emptyDrops method in DropletUtils, and gene expression matrices were generated using PISA.

### 2.15 Serum Biomarker Analysis

Serum samples were collected from a cohort comprising 35 bladder cancer patients and 16 healthy individuals. Circulating cell‐free DNA (cfDNA) was isolated from serum using the QIAamp ccfDNA/RNA Kit (55184, Qiagen, Germany) following the manufacturer's protocol. The concentration of purified cfDNA was subsequently determined using the Qubit dsDNA HS Assay Kit (Q32854, Invitrogen, USA). Serum concentrations of LRG1 (RK01800, ABclonal, China), as well as the NETosis‐associated markers Myeloperoxidase (MPO; RK00310, ABclonal, China) and Neutrophil Elastase (NE; RK00694, ABclonal, China), were determined using commercial ELISA kits. All assays were performed in duplicate, and optical density was measured at 450 nm using a microplate reader.

### 2.16 Evaluation of Mitochondrial Function and Abundance

The mitochondrial membrane potential (MMP) was evaluated using the JC‐1 (K2002, ApexBio, USA) and TMRE (C2001S, Beyotime, China) assay kits. Cells were loaded with the respective probes at 37°C for 20 min, and depolarization was monitored via fluorescence shifts or intensity changes. To assess relative mitochondrial mass and abundance, cells were stained with MitoTracker Deep Red FM (M22426, Thermo Fisher, USA) according to the manufacturer's guidelines. All corresponding images were acquired using a fluorescence microscope.

For the detection of mitochondrial superoxide, cells were incubated with the MitoSOX probe (A1678578, Ambeed, USA) at 37°C in the dark for 10 min. Nuclei were counterstained with Hoechst 33342 (C0031, Solarbio, China) for 5 min. The fluorescence signals were subsequently captured utilizing a fluorescence microscope.

Intracellular ATP levels were quantified employing an ATP Assay Kit (S0027, Beyotime, China). Briefly, cell lysates were subjected to centrifugation at 12,000 × *g* for 5 min at 4°C. The resulting supernatants were collected and mixed with a luciferase‐based working solution. Luminescence intensity was then recorded on a Tecan Infinite E plex multimode reader (Tecan, Switzerland) to determine the relative ATP concentrations.

Quantification of MitoSOX, Mitotracker, and TMRE staining was performed using ImageJ software. For each biological replicate across three independent experiments, relative fluorescence intensities were calculated based on at least five random fields containing 50–100 cells per field.

### 2.17 Animal Model

All animal experiments were approved by the Laboratory Animal Ethical Committee of Qilu Hospital of Shandong University (Approval No. DWLL‐202500043) and were conducted in strict accordance with the approved protocols, institutional guidelines, and relevant national regulations for the care and use of laboratory animals. C57BL/6 mice (8 weeks old) were housed in a specific pathogen‐free (SPF) facility under controlled temperature (22 ± 2°C) and a 12‐h light/dark cycle. In accordance with the ethical guidelines, the maximal permissible subcutaneous tumor size/burden was restricted to 1,500 mm^3^ in volume. We strictly confirm that this maximal tumor size/burden was not exceeded in any of the experimental animals throughout the course of this study. Mice were closely monitored and humanely euthanized immediately upon reaching predefined ethical endpoints or if any signs of severe morbidity or distress were observed.

#### 2.17.1 Tumor Models

For the subcutaneous model, MB49 cells (1 × 10^6^) suspended in 100 µl PBS were injected into the right inguinal region. Tumor volume was calculated as V = (length × width^2^) / 2.

For the orthotopic bladder cancer model, mice received a bladder injection of MB49 cells (1 × 10^6^ in 10 µl PBS) via catheterization.

For the lung metastasis model, MB49 cells (1 × 10^6^ in 100 µl PBS) were injected intravenously into the tail vein.

#### 2.17.2 Therapeutic Interventions

Neutrophil depletion: To deplete neutrophils, mice bearing Vector‐ or LRG1‐OE‐transduced MB49 tumors were treated with anti‐Ly6G antibody (400 µg initial dose, followed by 200 µg every 3 days; BE0075‐1, Bio X Cell, USA) or IgG2a isotype control (BE0089, Bio X Cell, USA). Treatment commenced one day prior to tumor inoculation.

NETs modulation: To induce NET formation, mice received an intraperitoneal (i.p.) injection of lipopolysaccharide (LPS; 10 µg/mouse; L8880S, Solarbio, China). For NET degradation, DNase 1 (100 U/mouse) was administered daily. In the LPS‐induced model, DNase 1 treatment began 24 h prior to LPS injection, followed by subcutaneous inoculation of MB49 cells 6 h post‐LPS [12]. In the LRG1‐OE model, DNase 1 was injected daily to eliminate NETs.

Cisplatin treatment: LRG1^+/+^ and LRG1^−/−^ mice were treated with cisplatin (2.5 mg/kg, i.p.) or vehicle every other day starting from day 5.

PD‐1 blockade: Mice were treated with anti‐PD‐1 antibody (200 µg; YR0292, ABclonal, China) or rat IgG2a isotype control (BE0089, Bio X Cell, USA) via i.p. injection every 3 days starting from day 3.

Tumors were harvested at different predefined endpoints depending on the specific experimental cohort to strictly comply with institutional ethical guidelines regarding maximum tumor burden. Specifically, tumors were harvested at day 15 post‐inoculation for the global LRG1^−/−^ host experiments, and at day 9 post‐inoculation for the LRG1‐overexpression and pharmacological treatment cohorts due to the accelerated tumor growth in these experimental groups.

### 2.18 Statistical Analysis

Statistical analyses were conducted using GraphPad Prism 9.0 and R. Data are expressed as mean ± SD from independent biological replicates. Differences between the two groups were assessed using Student's *t*‐test or the Mann‐Whitney U test, depending on the normality of the data. For multiple comparisons, one‐way ANOVA with Tukey's post hoc test was applied. Correlation analyses were performed using Spearman coefficients. Statistical significance was defined as *p* < 0.05.

## 3 Result

### 3.1 LRG1 Overexpression Correlates With Vascular Abnormalities and Hematogenous Metastasis in BCa

Integrated analysis of TCGA and GEO datasets (GSE188715/GSE13507) revealed significantly elevated LRG1 expression in BCa tissues (Figure 1A), with TCGA data further demonstrating a gradient upregulation across advancing T stages (Figure 1B). To further dissect the cellular origins and heterogeneity of LRG1 expression within the tumor microenvironment, we integrated four public single‐cell RNA sequencing (scRNA‐seq) datasets (GSE301651, GSE267718, GSE225190, and GSE175526), comprising 5 normal bladder and 19 BCa samples. Unsupervised clustering successfully annotated all cells into 10 distinct cell types (Figure 1C). Comparative analysis between normal epithelial cells and malignant cells revealed that LRG1 expression was significantly upregulated in the malignant compartment (Figure 1D). To parse the functional diversity of these malignant cells, we applied Non‐negative Matrix Factorization (NMF) to their transcriptomic profiles. By scoring and clustering based on meta‐program signature genes, we identified 6 distinct tumor subclusters (Figure 1E), among which the “LRG1” subcluster exhibited remarkably high LRG1 expression. Subsequently, utilizing the top marker genes from these subclusters, we evaluated their prognostic impact in the bulk RNA‐seq cohorts. Kaplan‐Meier survival analysis demonstrated that high expression of the LRG1 meta‐program was significantly associated with poorer overall survival in the TCGA and GSE13507 cohorts (Figure 1F), although no significant impact was observed in the GSE188715 dataset (data not shown).

**FIGURE 1:**
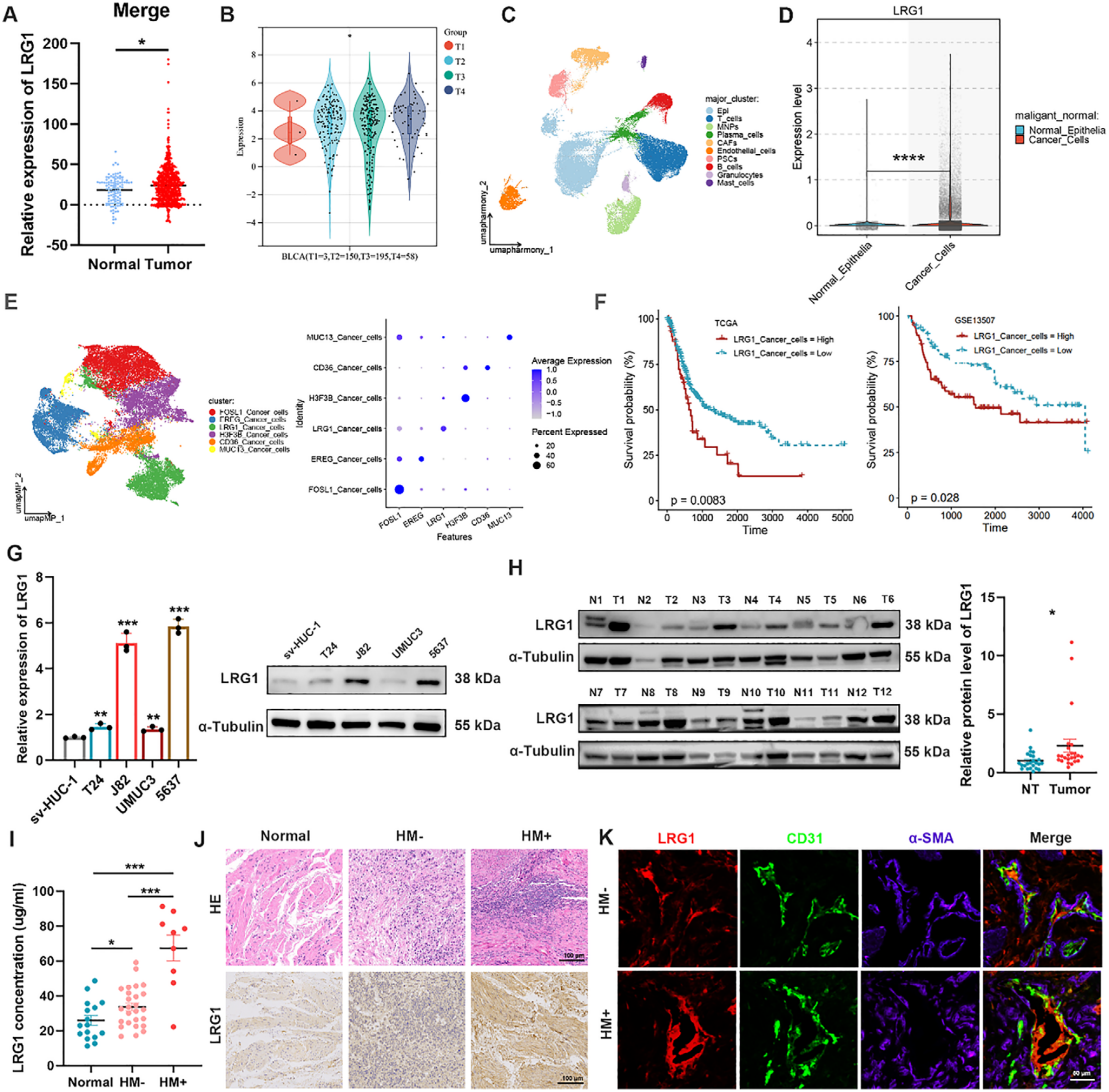
LRG1 is upregulated in bladder cancer and correlates with hematogenous metastasis and vascular abnormalities. (A, B) Differential expression of LRG1 in BCa versus normal tissues using merged data from the TCGA and GEO (GSE188715 and GSE13507) datasets (A), and its correlation with T‐stages analyzed exclusively from the TCGA cohort (B). (C) UMAP plot annotating 10 distinct cell types from the integrated scRNA‐seq BCa datasets (GSE301651, GSE267718, GSE225190, and GSE175526). (D) Comparative analysis showing elevated LRG1 expression in malignant tumor cells versus normal epithelial cells. (E) NMF‐based subclustering of malignant cells into 6 distinct tumor meta‐programs, highlighting the LRG1‐high subcluster. (F) Kaplan‐Meier survival curves evaluating the prognostic impact of the LRG1 meta‐program marker genes in the TCGA and GSE13507 cohorts. (G, H) Validation of LRG1 expression levels in BCa cell lines via qRT‐PCR and Western blotting (C), and in paired clinical BCa specimens via Western blotting (D). (I, J) ELISA quantification of serum LRG1 concentrations (E) and representative H&E and IHC images of LRG1 expression (F) in patients with or without hematogenous metastasis (HM+ vs. HM‐). (K) Representative confocal multiplex immunofluorescence images showing the spatiotemporal colocalization of LRG1 (red), endothelial marker CD31 (green), and mural cell marker α‐SMA (blue) in BCa tissues.

Prompted by these profound transcriptomic and clinical prognostic correlations, we next sought to experimentally verify LRG1 overexpression. Robust LRG1 upregulation was consistently confirmed in BCa cell lines and paired clinical specimens (Figure 1G,H). Clinically, LRG1 abundance was closely associated with hematogenous metastasis (HM). Serum LRG1 concentrations were markedly higher in HM+ patients than in HM‐ patients or healthy controls (Figure 1I). Consistently, histological examination (HE and IHC) demonstrated robust LRG1 accumulation in HM+ tumor tissues compared to HM‐ and normal tissues (Figure 1J; Figure S1B). Furthermore, immunofluorescence staining for LRG1, CD31, and α‐SMA revealed a distinct spatiotemporal colocalization of LRG1 with the endothelial marker CD31 (Figure 1K). Notably, LRG1‐enriched HM+ tissues exhibited reduced pericyte coverage (α‐SMA), strongly suggesting that LRG1 preferentially accumulates in the vascular niche to compromise vascular integrity and facilitate metastasis (Figure S1C).

### 3.2 Genetic Ablation of Lrg1 Suppresses BCa Progression and Promotes Tumor Vessel Normalization

To investigate the functional role of LRG1 in vivo, we established an Lrg1 knockout mouse model and evaluated the growth of MB49 bladder cancer cells. In the subcutaneous xenograft model, Lrg1 deficiency significantly suppressed tumor growth (Figure 2A). This tumor‐suppressive effect was further confirmed in the orthotopic bladder cancer model, demonstrating a restricted tumor burden in LRG1^−/−^ mice (Figure 2B,C). Furthermore, necropsy of major organs revealed no observable macroscopic abnormalities or systemic toxicity associated with LRG1 knockout (Figure S2A). Given the clinical correlation between LRG1 and hematogenous metastasis, we next assessed the impact of Lrg1 ablation on tumor dissemination using a tail vein injection model. Strikingly, lung metastases were drastically attenuated in LRG1^−/−^ mice compared to LRG1^+/+^ mice (Figure 2D,E).

**FIGURE 2:**
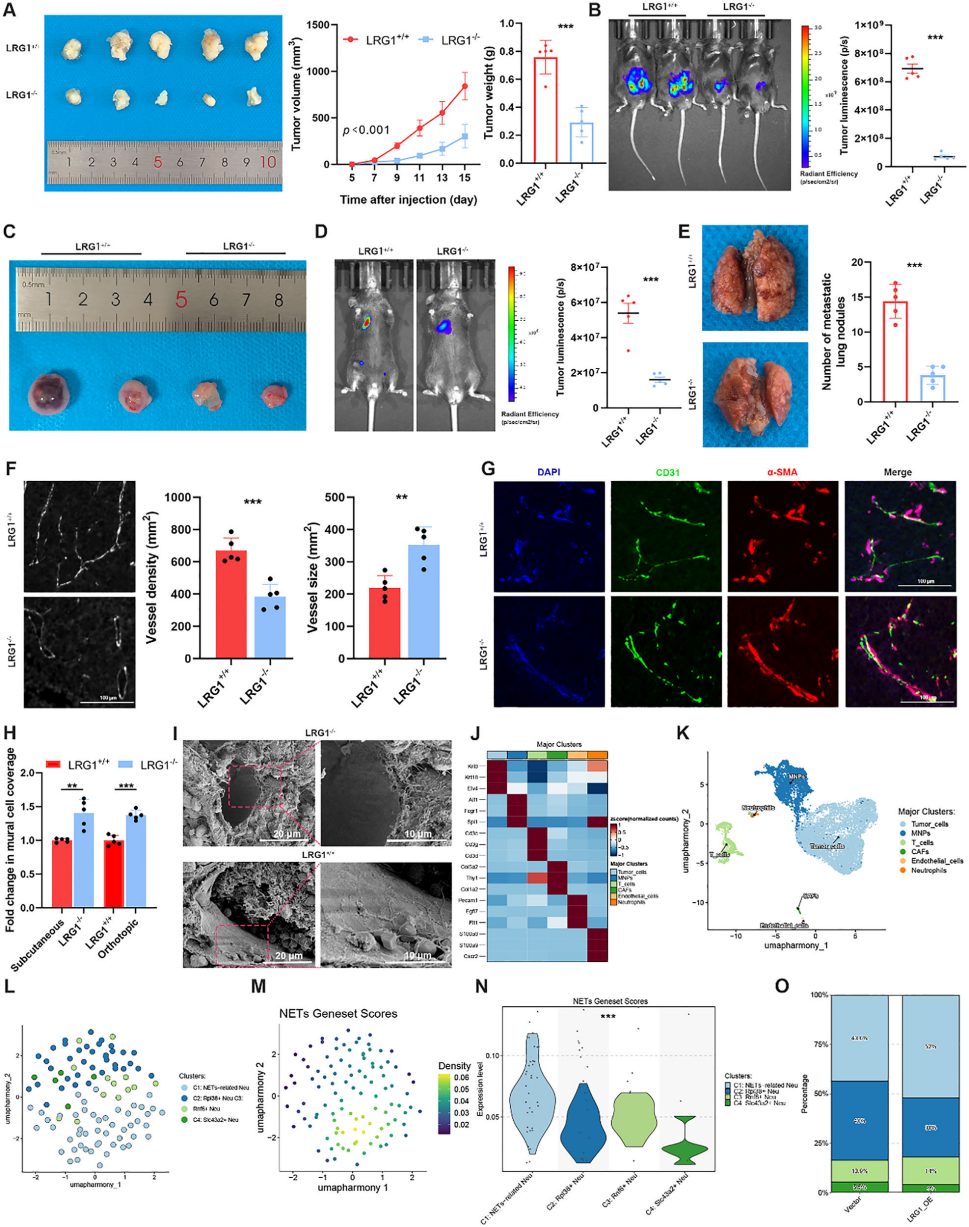
Genetic ablation of Lrg1 restrains tumor progression, normalizes vasculature, and reshapes the immune microenvironment. (A) Tumor growth curves, macroscopic anatomical images, and endpoint tumor weights of wild‐type (LRG1^+/+^) and LRG1 knockout (LRG1^−/−^) subcutaneous MB49 tumor models. (B, C) Representative bioluminescence images (B) and macroscopic anatomical observations (C) of orthotopic bladder tumors in LRG1^+/+^ and Lrg1^−/−^ mice. (D, E) Representative bioluminescence images with corresponding statistical analysis (D), and macroscopic anatomical images of the lungs detailing metastatic nodules along with their quantification (E) in the experimental tail‐vein injection model. (F) Immunofluorescence staining for CD31 and quantitative analysis of overall microvessel density and vessel diameter. (G, H) Representative confocal double immunofluorescence images for CD31 (green) and α‐SMA (red) (G) in subcutaneous tumor models and quantitative analysis of pericyte coverage (H) in both subcutaneous and orthotopic tumor models. (I) Scanning electron microscopy (SEM) images displaying the ultrastructure of tumor vascular endothelium and intraluminal membranous inclusions. (J) Expression profiles of canonical marker genes across different cell clusters, with red indicating high expression and blue indicating low expression. (K) Uniform Manifold Approximation and Projection (UMAP) plot displaying the distribution of major cell lineages in the mouse BCa tumor tissues, color‐coded by distinct clusters. (L) UMAP plot demonstrating the subclustering of neutrophils into four distinct subpopulations (C1‐C4). (M) UCell functional enrichment scoring of the NETs signature gene set within the neutrophil populations, where brighter colors represent higher enrichment scores. (N) Violin plots quantifying the NETs gene set scores across the neutrophil subpopulations. (O) Comparative relative proportions of the four neutrophil subpopulations between the LRG1‐OE and WT groups.

To determine whether the restricted tumor growth and metastasis in LRG1^−/−^ mice were linked to vascular alterations, we investigated the effect of Lrg1 on angiogenesis. Immunofluorescence staining and confocal microscopy for CD31 revealed that within the MB49 tumor tissues, Lrg1 knockout significantly decreased overall microvessel density but enlarged vessel diameter, a morphological hallmark of vascular normalization [13] (Figure 2F). Furthermore, double immunofluorescence staining for CD31 and α‐SMA showed a consistent decrease in pericyte coverage across both subcutaneous and orthotopic tumors in the LRG1^−/−^ cohort (Figure 2G,H; Figure S2B). Moreover, Laminin immunofluorescence revealed that the highly fragmented and disorganized vascular structures typical of control tumors were remodeled into continuous and uniform layers following LRG1 ablation (Figure S2C), indicating restored basement membrane integrity. To further substantiate the role of LRG1 in vascular architectural integrity, scanning electron microscopy of MB49 tumors revealed a pronounced reduction in intraluminal endothelial inclusions in LRG1^−/−^ mice (Figure 2I), confirming the amelioration of pathological and leaky tumor microvessels. Collectively, these findings indicate that the genetic ablation of Lrg1 not only restrains BCa growth and dissemination but also promotes tumor vessel normalization.

### 3.3 Single‐Cell Transcriptomics Reveals an LRG1‐Driven Enrichment of NET‐Forming Neutrophils in the TME

Given the profound impact of LRG1 on the tumor vasculature and metastasis, we hypothesized that LRG1 might orchestrate these effects by reshaping the TME. To comprehensively profile the TME landscape, we performed scRNA‐seq on subcutaneous tumor tissues derived from control and LRG1‐OE MB49 cells (Figure S3A). Following stringent quality control to remove low‐quality cells and doublets, a total of 13,454 high‐quality cells were retained for downstream analysis. Unsupervised clustering identified six major distinct cell lineages (Figure 2J), which were annotated based on canonical marker genes as tumor cells, mononuclear phagocytes (MNPs), T cells, cancer‐associated fibroblasts (CAFs), endothelial cells, and neutrophils (Figure 2K). Notably, the neutrophil compartment exhibited significant transcriptomic shifts between the experimental groups. To dissect neutrophil heterogeneity, we extracted this population and performed subclustering, revealing four distinct neutrophil subpopulations (C1–C4) (Figure 2L). Functional enrichment scoring using the UCell method highlighted that the C1 subpopulation highly expressed a signature gene set associated with neutrophil extracellular traps (NETs) (Figure 2M; Table S3). Statistical analysis of the NETs gene set scores confirmed that the C1 subpopulation possessed a significantly higher NET‐forming potential than the other subclusters (*p* < 0.001, Figure 2N). Consequently, we defined the C1 subcluster as “NETs‐related neutrophils”. Crucially, comparative analysis of subpopulation proportions revealed that the infiltration of NETs‐related neutrophils was markedly enriched in the LRG1‐OE tumors compared to the WT group (Figure 2O). This finding strongly suggests that LRG1 expression is closely linked to the induction of NETosis within the TME.

### 3.4 LRG1 Promotes Bladder Cancer Progression and Metastasis by Recruiting Neutrophils and Inducing NETosis

Clinical analysis of patient samples revealed that the infiltration density of CD66b+ neutrophils in HM+ bladder cancer tissues was significantly higher than that in non‐metastatic and paracancerous tissues (Figure 3A,B). Consistent with these findings, pooled dataset analysis indicated that high neutrophil infiltration correlated with poorer survival outcomes (Figure S3B). Furthermore, immune infiltration analysis of the TCGA database using both CIBERSORT and MCPcounter algorithms corroborated that LRG1 expression levels were positively correlated with neutrophil abundance (Figure S3C–F).

**FIGURE 3:**
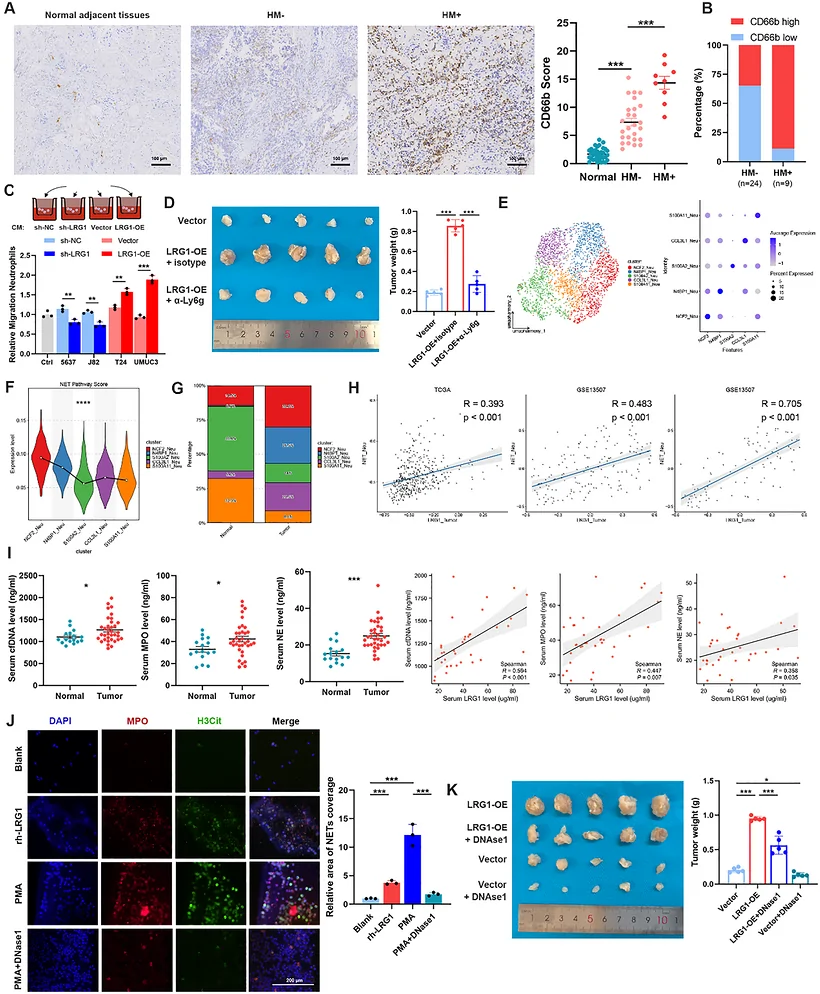
Tumor‐derived LRG1 promotes cancer progression by recruiting neutrophils and driving NETosis. (A, B) Representative IHC images and quantification of CD66b+ neutrophil infiltration density in HM+ versus HM‐ BCa tissues. (C) Transwell assays evaluating the chemotactic effect of tumor‐derived LRG1 on isolated human neutrophils. (D) Representative macroscopic images and endpoint tumor weights demonstrating the “neutrophil‐dependent” tumor‐promoting effect of LRG1 in vivo, which is effectively rescued by anti‐Ly6G antibody depletion. (E) UMAP plot demonstrating the reclustering and annotation of distinct neutrophil subpopulations based on subcluster‐specific marker genes from the scRNA‐seq cohort. (F) Gene set enrichment scoring of the KEGG NETs pathway across neutrophil subpopulations, highlighting significant enrichment in the NCF2_neu subset. (G) Comparative analysis of the relative proportion of the NCF2_neu subpopulation between normal and BCa tumor tissues. (H) GSVA correlation analysis between the LRG1_tumor meta‐program and NCF2_neu infiltration abundance across three independent bulk RNA‐seq datasets. (I) ELISA quantification of serum NETs markers (cfDNA, MPO, NE) and their correlation with LRG1 levels. (J) Immunofluorescence staining for MPO and H3Cit in neutrophils directly stimulated with rh‐LRG1 or PMA. (K) Representative macroscopic images and endpoint tumor weights demonstrating the rescue of LRG1‐induced tumor progression by DNase I treatment in vivo.

To functionally delineate the impact of tumor‐derived LRG1 on neutrophil behavior in vitro, we freshly isolated primary human neutrophils from the peripheral blood of healthy volunteers. The high purity of the isolated neutrophils (>95%) was robustly verified via Wright–Giemsa staining and flow cytometric analysis (Figure S3G,H), ensuring the reliability of downstream functional assessments. Next, we established LRG1‐OE T24 and UMUC3 cell lines, confirming a significant increase of LRG1 in the supernatant, alongside LRG1‐knockdown 5637 and J82 cell lines (Figure S3I). To rigorously exclude the possibility that LRG1‐induced neutrophil accumulation is a mere consequence of altered cell survival, we evaluated neutrophil apoptosis following incubation with tumor‐derived CM. Flow cytometric analysis revealed that the LRG1‐OE CM did not significantly affect the baseline apoptotic rate of neutrophils (Figure S3J). Having ruled out survival biases, we assessed the chemotactic effects of the CM. Results demonstrated that LRG1 overexpression significantly enhanced the ability of CM to attract neutrophils, whereas LRG1 knockdown exerted the opposite effect (Figure 3C). Mechanistically, we observed that recombinant human LRG1 (rhLRG1) could internalize into neutrophils via the lysosomal pathway (Figure S3K). Furthermore, in vitro co‐culture assays revealed that the presence of neutrophils significantly amplified the pro‐angiogenic effects of rhLRG1 on endothelial cells, underscoring a synergistic interaction between LRG1 and recruited neutrophils in driving vascular remodeling (Figure S3L). To determine whether neutrophils are the indispensable mediators of LRG1‐induced tumor promotion, we depleted neutrophils in vivo using an α‐Ly6g antibody. This depletion effectively abolished the tumor‐promoting effect of LRG1‐overexpressing MB49 cells (Figure 3D), suggesting that neutrophils play a pivotal role in LRG1‐driven oncogenesis.

To gain deeper mechanistic insights into the functional heterogeneity of these recruited neutrophils, we revisited our scRNA‐seq data. Neutrophils were extracted and reclustered, allowing us to annotate distinct neutrophil subpopulations based on their specific marker genes (Figure 3E). To evaluate their capacity for NETosis, we performed gene set scoring utilizing the KEGG NETs pathway signature. This analysis revealed that the NETs pathway was significantly enriched in the NCF2+ neutrophil (NCF2_neu) subpopulation (Figure 3F). Consequently, we defined NCF2_neu as the NET‐associated neutrophil subset. Notably, the relative proportion of NCF2_neu cells was substantially expanded in tumor tissues compared to adjacent normal tissues (Figure 3G). To further establish the spatial and functional correlation between LRG1‐expressing tumor cells and this specific neutrophil subset, we performed Gene Set Variation Analysis (GSVA) across three independent bulk RNA‐seq datasets to deconvolute their relative infiltration abundance. Intriguingly, we observed a profound positive correlation between the LRG1_tumor meta‐program and NCF2_neu abundance (Figure 3H), indicating a highly specific recruitment and programming axis.

Beyond in silico correlations and general recruitment, we sought to determine if LRG1 directly modulates the functional state of these neutrophils to induce NETosis. Serological testing showed that the concentrations of Neutrophil Extracellular Traps (NETs)‐associated markers (cfDNA, MPO, and NE) were significantly elevated in the circulation of bladder cancer patients compared to healthy controls. Correlation analysis confirmed a strong positive relationship between LRG1 levels and these markers, particularly cfDNA (r = 0.59, Figure 3I). Crucially, direct stimulation of neutrophils with rhLRG1 significantly upregulated the expression of NETs markers MPO and H3Cit (Figure 3J). This evidence confirms that LRG1 acts not only as a chemoattractant but also as a potent inducer of NETosis. Finally, in vivo studies showed that the increased tumor volume and weight observed in LRG1‐overexpressing tumors were significantly counteracted by the administration of DNase 1 (Figure 3K), thereby establishing the LRG1‐Neutrophil‐NETosis axis as a critical driver of bladder cancer progression.

### 3.5 NETs Promote Bladder Cancer Progression and Destabilize Tumor Vasculature

To investigate the direct functional role of NETs in tumor progression, we first established a robust in vitro NETosis model. SYTOX Green staining visually confirmed the massive release of extracellular DNA webs from neutrophils following PMA stimulation, which were efficiently dismantled upon DNase I treatment (Figure S3M). While NETs are well‐documented to physically ensnare pathogens and have been reported to trap circulating tumor cells in multiple malignancies, whether this “trapping” effect occurs specifically in BCa cells remains to be elucidated. To validate this in BCa, we performed in vitro neutrophil adhesion assays. The results revealed that BCa cells were densely anchored and trapped within the extensive NET networks. Crucially, targeted degradation of these DNA scaffolds with DNase I completely abolished this heterotypic adhesion (Figure 4A), confirming that NETs indeed possess the capacity to physically entrap BCa cells. Beyond physical capturing, Transwell co‐culture assays demonstrated that exposure to intact NETs significantly augmented the migratory capacity of BCa cells (Figure 4B).

**FIGURE 4:**
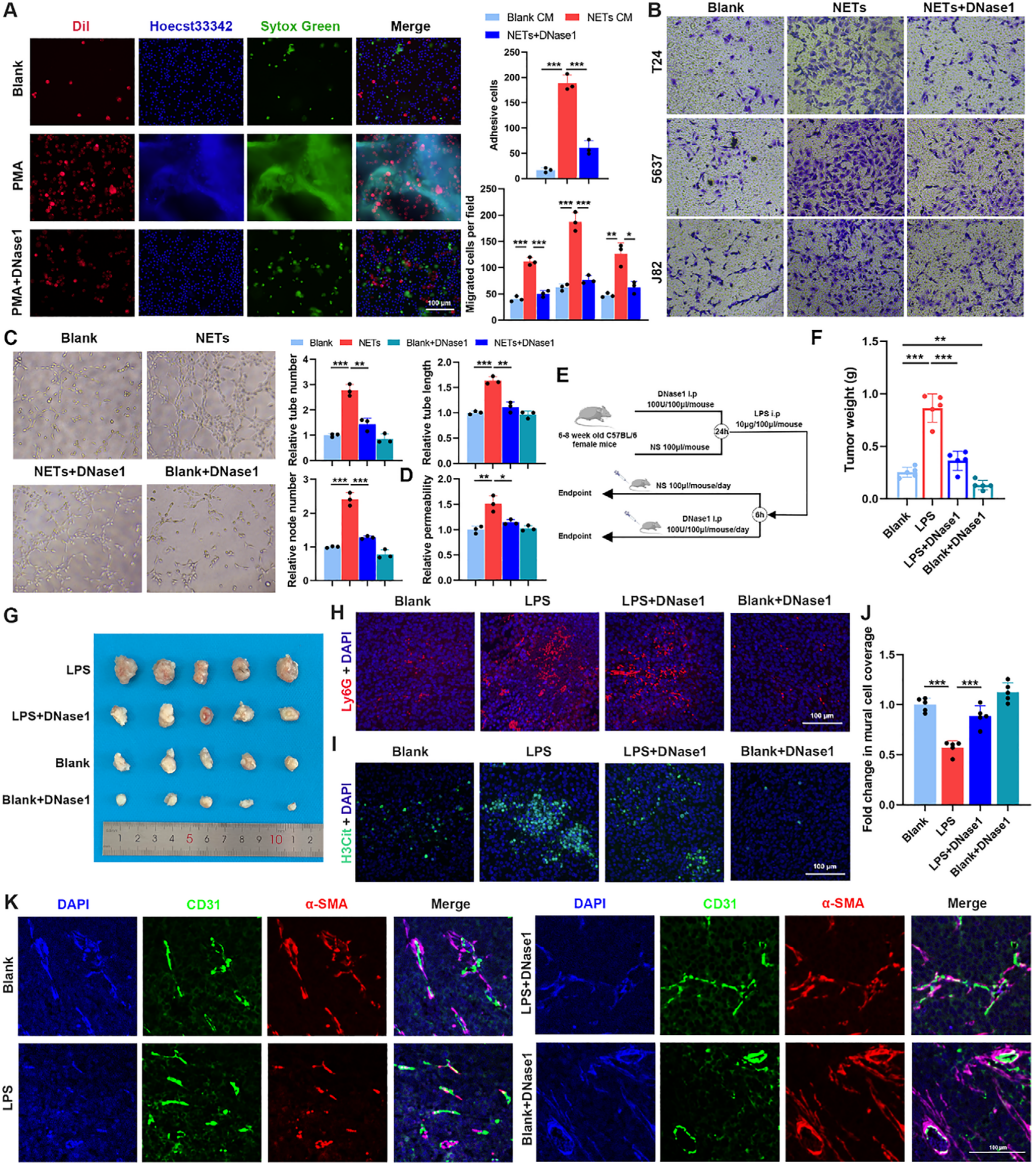
NETs facilitate tumor cell trapping and directly destabilize tumor vasculature. (A) In vitro neutrophil adhesion assay evaluating the trapping effect of NETs on BCa cells and the reversal by DNase I. (B) Transwell co‐culture assay evaluating the impact of intact NETs on the migratory capacity of BCa cells. (C) In vitro tube formation assay utilizing HUVECs to assess the pro‐angiogenic capacity of NETs. (D) Endothelial permeability assay measuring TRITC‐Dextran flux across HUVEC monolayers following NETs stimulation. (E) Schematic timeline of the in vivo LPS‐induced systemic NETosis model and DNase I intervention. (F, G) Endpoint tumor weights (F) and macroscopic images (G) of subcutaneous tumors from respective groups. (H, I) Representative immunofluorescence images depicting neutrophil recruitment (Ly6G, H) and NETs formation (H3Cit, I) in tumor tissues. (J, K) Quantitative analysis of mural cell coverage (J) and representative confocal dual immunofluorescence images for CD31/α‐SMA (K) to confirm NET‐induced vascular destabilization.

Our preceding data demonstrated that LRG1 overexpression leads to both reduced mural cell coverage and robust NETosis. This prompted us to hypothesize that NETs might act as the direct downstream effectors mediating LRG1‐induced vascular destabilization and pathological angiogenesis. Supporting this clinical and spatial relevance, our GSVA‐based deconvolution across bulk RNA‐seq datasets revealed a significant positive correlation between the infiltration abundance of the NET‐associated NCF2_neu subpopulation and endothelial cell signatures (Figure S3N). To experimentally test this, we evaluated the direct impact of NETs on endothelial cells. In vitro tube formation assays revealed that NETs remarkably accelerated angiogenesis, as evidenced by significant increases in total tube length, the number of capillary‐like structures, and junctional nodes (Figure 4C). Furthermore, since vascular destabilization is often characterized by compromised endothelial barrier function, we performed permeability assays. The results showed that NETs directly induced hyperpermeability in endothelial monolayers (Figure 4D), creating a leaky microenvironment that favors cancer cell extravasation.

To substantiate these findings in vivo, we employed an LPS‐induced systemic NETosis model in C57BL/6 mice subcutaneously inoculated with MB49 cells. As expected, LPS administration significantly accelerated tumor growth. To rigorously rule out the confounding inflammatory effects of LPS and isolate the specific contribution of NETs, we simultaneously administered DNase I to degrade the circulating and tumor‐deposited NETs. Strikingly, DNase I treatment profoundly abrogated the LPS‐induced tumor‐promoting effects (Figure 4E–G). Immunofluorescence profiling of the tumor microenvironment further corroborated this mechanism: Ly6G staining confirmed the massive recruitment of neutrophils upon LPS challenge (Figure 4H; Figure S3O), while H3Cit staining verified the robust induction of NETs, which was effectively cleared by concurrent DNase I therapy (Figure 4I; Figure S3P). Finally, to evaluate in vivo vascular stability, we conducted dual immunofluorescence staining for CD31 and α‐SMA. Confocal imaging definitively showed that LPS‐induced NETs accumulation severely diminished mural cell coverage, a profound vascular destabilization that sharply contrasts with the “vascular normalization” phenotype we previously observed in Lrg1‐deficient tumors; conversely, targeted degradation of NETs by DNase I significantly restored pericyte coverage and vascular integrity (Figure 4J,k).

### 3.6 LRG1 Directly Interacts With ANXA2 via Its Leucine‐Rich Repeat (LRR) Domain

Initial transcriptomic profiling via RNA sequencing (RNA‐seq) on primary neutrophils revealed that LRG1 stimulation orchestrates a profound intracellular reprogramming (Figure S4A). Gene Ontology (GO) and KEGG enrichment analyses revealed a profound transcriptomic reprogramming (Figure S4B,C). Specifically, LRG1 stimulation significantly enriched biological processes and pathways associated with chromatin remodeling (e.g., histone‐modifying activity), intracellular dynamics (organelle transport along microtubules), and crucial metabolic shifts, particularly oxidative phosphorylation and nucleotide metabolism. Notably, immune‐regulatory networks, including the PD‐L1 and PD‐1 checkpoint pathway, were also highly highlighted. Driven by these extensive intracellular alterations, we next sought to identify the direct downstream interacting targets mediating the LRG1 signal.

To elucidate the molecular mechanisms underlying LRG1‐mediated NETosis, neutrophils were first treated with His‐tagged recombinant human LRG1 (rhLRG1). Subsequently, we performed pull‐down assays using either anti‐LRG1 or anti‐His tag antibodies, followed by high‐sensitivity silver staining and mass spectrometry analysis to map the LRG1 interactome (Figure 5A). The results revealed that annexin A2 (ANXA2) was significantly enriched in both the anti‐LRG1 and anti‐His pull‐down groups compared to the IgG control, indicating that it is a direct interacting target of LRG1 (Figure 5A,B). This physical interaction was subsequently validated through endogenous and exogenous co‐immunoprecipitation (Co‐IP) assays in primary human neutrophils and HEK‐293T cells, respectively (Figure 5C,D). Of note, LRG1 exhibited varying molecular weights across these assays—appearing at 55 kDa when derived from neutrophils and 38 kDa from tumor cells or tissues—a discrepancy primarily attributed to differential glycosylation levels [14].

**FIGURE 5:**
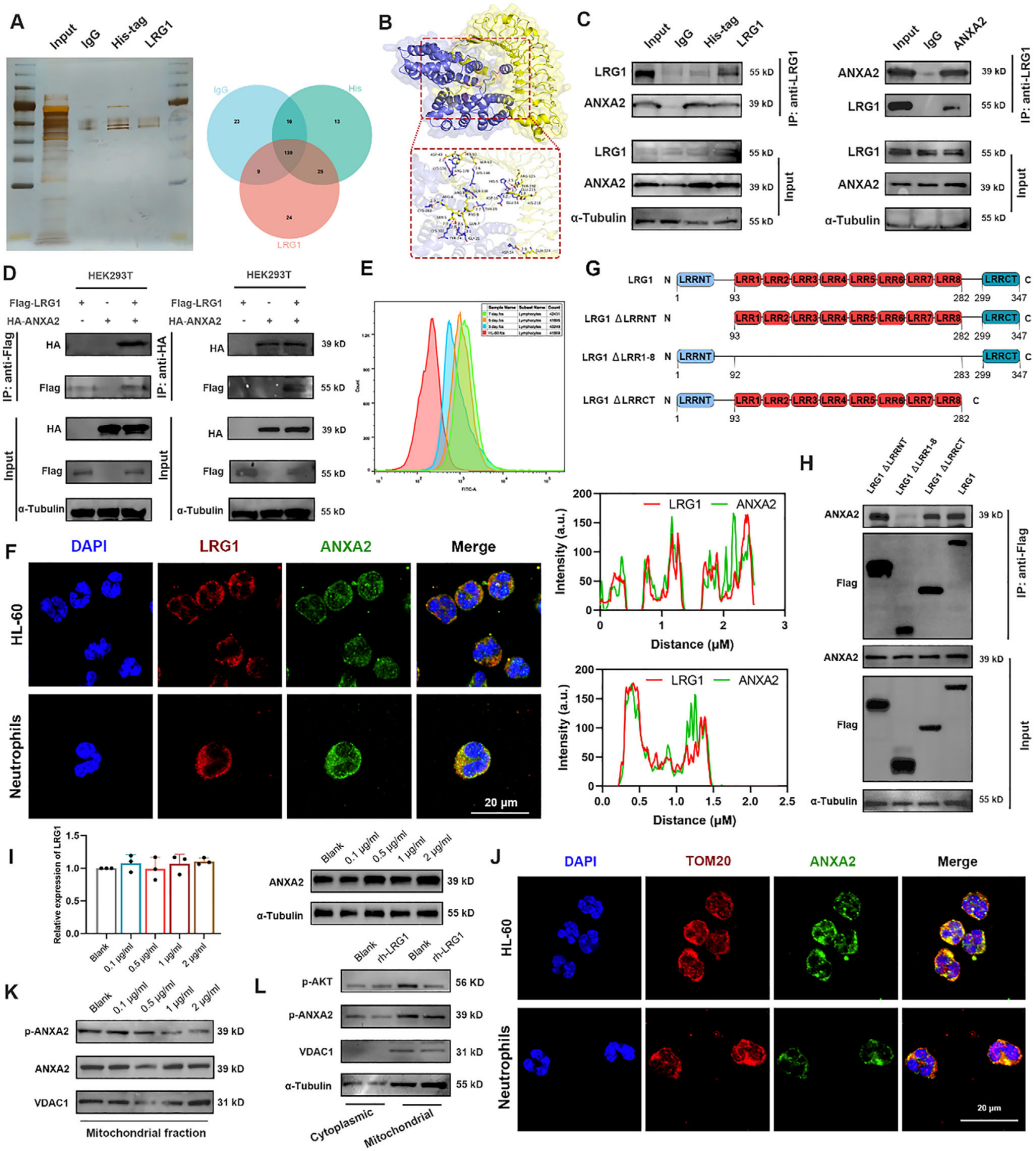
LRG1 directly interacts with ANXA2 via its LRR domain and impedes the mitochondrial translocation of active Akt. (A) Silver staining and Venn diagram of mass spectrometry analysis identifying ANXA2 as a direct interactor following pull‐down assays with anti‐LRG1 or anti‐His antibodies. (B) Structural binding mode diagram illustrating the molecular interaction interface between LRG1 (yellow) and ANXA2 (blue). (C, D) Endogenous (C, neutrophils) and exogenous (D, HEK‐293T) Co‐IP assays validating the physical interaction between LRG1 and ANXA2. (E) Flow cytometric validation of CD11b expression in DMSO‐differentiated HL‐60 (dHL‐60) cells. (F) Confocal microscopy demonstrating the colocalization of LRG1 and ANXA2 in the cytoplasm of primary neutrophils and dHL‐60 cells. (G, H) Schematic of LRG1 truncation mutants (G) and Co‐IP assays (H) pinpointing the LRR1‐8 domain as essential for ANXA2 binding. (I) qRT‐PCR and Western blot analysis of ANXA2 expression following LRG1 stimulation. (J) Immunofluorescence confirming the colocalization of ANXA2 with the mitochondrial marker TOM20. (K, L) Immunoblotting of isolated mitochondrial fractions showing the suppressive effect of LRG1 on ANXA2 phosphorylation (K) and the concurrent reduction of mitochondrial pSer473‐Akt (L).

Mechanistic investigations in primary human neutrophils are inherently hindered by their terminally differentiated state, short ex vivo lifespan, and resistance to genetic manipulation. To circumvent these limitations, we utilized DMSO‐differentiated HL‐60 (dHL‐60) cells, a well‐established in vitro model for studying NET formation [15]. Flow cytometric analysis of the surface marker CD11b confirmed that the majority of HL‐60 cells successfully differentiated into neutrophil‐like cells following 5 days of DMSO induction (Figure 5E). Utilizing this optimized model alongside primary neutrophils, confocal microscopy demonstrated that LRG1 predominantly localizes to the cytoplasm, where it strongly colocalizes with ANXA2 (Figure 5F), further supporting their physiological interaction.

To delineate the specific structural regions responsible for the LRG1‐ANXA2 interaction, we generated a series of LRG1 truncation mutants to pinpoint the binding site (Figure 5G). Leucine‐rich repeat (LRR) domains typically form horseshoe‐shaped structures that are well‐recognized as ideal motifs for mediating protein‐protein interactions [16]. Consistent with this structural feature, our transfection experiments in HEK‐293T cells revealed that the deletion of the LRR1‐8 region (amino acids 93–282) completely abolished the ability of LRG1 to bind ANXA2. Conversely, the deletion of either the LRR N‐terminal (LRRNT) or C‐terminal (LRRCT) domain had no such detrimental effect (Figure 5H). Collectively, these data demonstrate that the central LRR domain of LRG1 is essential for its direct interaction with ANXA2.

### 3.7 LRG1 Abrogates ANXA2‐Mediated Mitochondrial Translocation of p‐AKT to Induce mtROS‐Dependent NETosis

Having established the physical interaction between LRG1 and ANXA2, we investigated the downstream molecular consequences. Notably, LRG1 stimulation did not significantly alter the overall mRNA or protein levels of ANXA2 (Figure 5I). Given that ANXA2 is a mitochondria‐associated protein whose biological function is tightly dictated by its phosphorylation status [17, 18]—particularly at the Tyrosine 24 (Tyr24) residue—we hypothesized that LRG1 might modulate its post‐translational modification. Immunofluorescence analysis first confirmed the spatial colocalization of ANXA2 with the mitochondrial marker TOM20 (Figure 5J; Figure S5A). Subsequent immunoblotting of isolated mitochondrial fractions revealed that rhLRG1 treatment drastically suppressed the phosphorylation of mitochondrial ANXA2 (Figure 5K). Previous studies have implicated ANXA2 in the regulation of Akt activation, functioning as a critical rheostat for the subcellular shuttling of active Akt (pSer473‐Akt) [19]. Subcellular fractionation analyses demonstrated that rhLRG1 treatment not only diminished mitochondrial p‐ANXA2 but concurrently abolished the mitochondrial accumulation of pSer473‐Akt, compared to the cytosolic fraction (Figure 5L).

To establish a causal link between ANXA2 Tyr24 phosphorylation and pSer473‐Akt mitochondrial translocation, we engineered ANXA2 phosphomimetic (Y24D) and phospho‐dead (Y24A) mutants (Figure 6A). Both Western blotting and IF analyses confirmed that pSer473‐Akt was robustly localized to the mitochondria in ANXA2‐Y24D expressing cells, whereas this mitochondrial compartmentalization was profoundly impaired in the ANXA2‐Y24A group (Figure 6B,C). Crucially, co‐immunoprecipitation assays revealed that rhLRG1 or LRG1‐OE CM effectively disrupted the physical interaction between p‐ANXA2 and p‐Akt within the mitochondria (Figure 6D). Collectively, these data indicate that LRG1 dismantles the ANXA2/Akt complex, thereby impeding both the phosphorylation‐dependent activation of Akt and its essential mitochondrial translocation.

**FIGURE 6:**
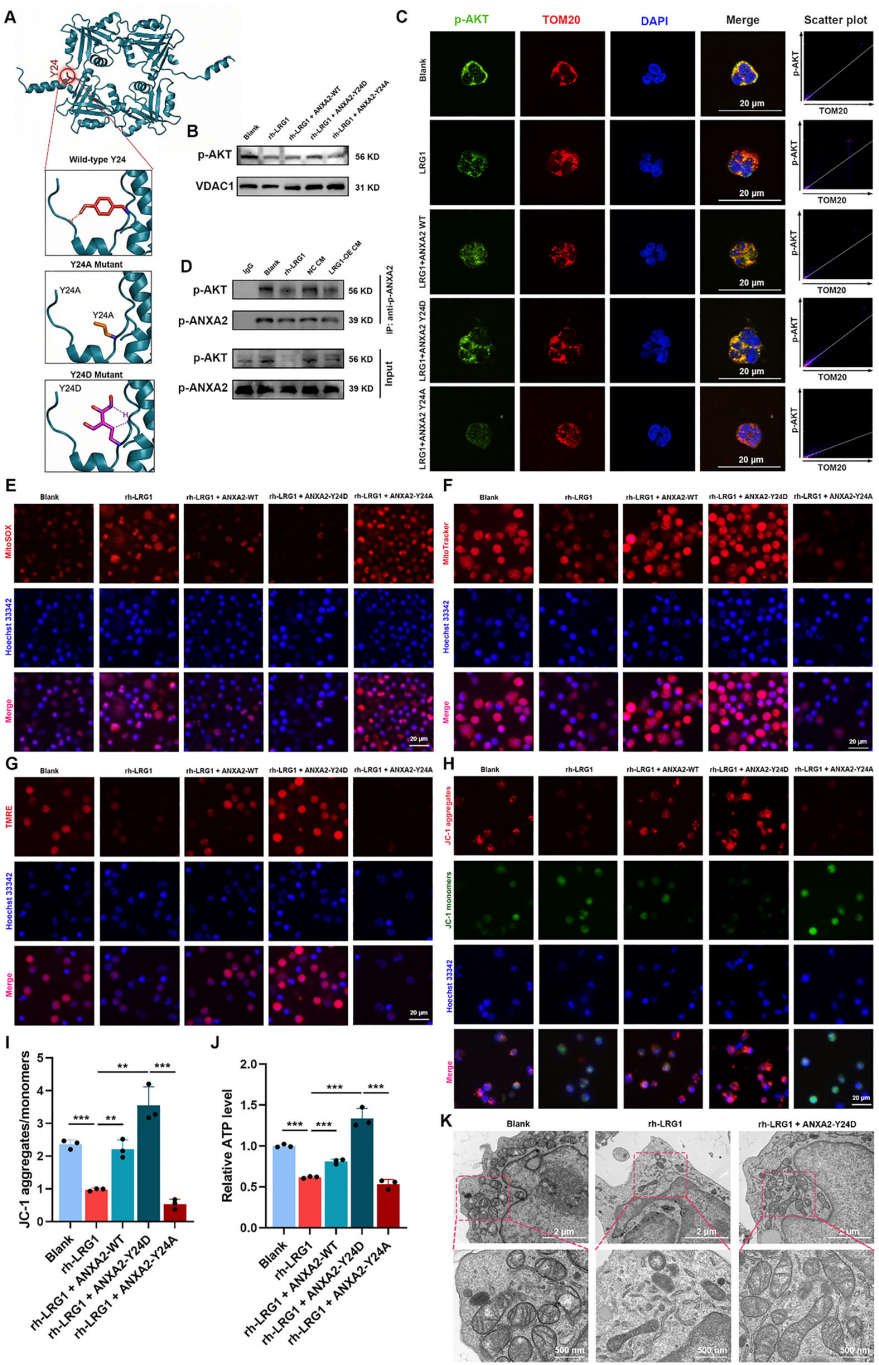
LRG1 triggers mtROS‐dependent NETosis by abrogating the ANXA2/Akt signaling axis. (A) Schematic representation of ANXA2 phosphomimetic (Y24D) and phospho‐dead (Y24A) mutant constructs. (B, C) Western blot (B) and confocal imaging (C) confirming the restorative effect of ANXA2‐Y24D on the mitochondrial localization of pSer473‐Akt. (D) Co‐IP assay indicating that LRG1 or LRG1‐CM disrupts the interaction between p‐ANXA2 and p‐Akt in the mitochondria. (E) Quantification of mtROS levels using the MitoSOX probe upon rhLRG1 stimulation and mutant rescue. (F) Assessment of mitochondrial mass utilizing MitoTracker staining across the corresponding experimental groups. (G–I) Fluorescence assessment of severe mitochondrial membrane depolarization utilizing TMRE and JC‐1 probes. (J) Measurement of relative intracellular ATP synthesis capacity across groups. (K) Transmission electron microscopy (TEM) images revealing LRG1‐induced ultrastructural aberrations (swelling, cristae disorganization) and their partial amelioration by the ANXA2‐Y24D mutant.

The suppression of mitochondrial p‐AKT signaling directly promotes the pathological accumulation of mtROS, which acts as a pivotal trigger for initiating NETosis [11]. We therefore assessed the impact of the LRG1/ANXA2 axis on neutrophil mitochondrial homeostasis. Compared to the blank control, rhLRG1 treatment triggered a massive surge in mtROS levels (Figure 6E; Figure S5B) and a concurrent reduction in mitochondrial mass (Figure 6F). This was accompanied by severe mitochondrial membrane depolarization (Figure 6G–I) and compromised ATP synthesis capacity (Figure 6J), signifying a catastrophic collapse of mitochondrial homeostasis. Importantly, the introduction of the ANXA2‐Y24D mutant substantially reversed these metabolic defects, whereas the ANXA2‐Y24A mutant exacerbated the LRG1‐induced mitochondrial failure. Finally, transmission electron microscopy provided direct ultrastructural evidence of this damage. Mitochondria in rhLRG1‐treated neutrophils exhibited notable structural aberrations, characterized primarily by electron‐dense matrices, overt organelle swelling, and the disorganization of mitochondrial cristae (Figure 6K). Consistent with our biochemical data, the ANXA2‐Y24D mutant partially ameliorated these ultrastructural deformities. Together, these findings compellingly demonstrate that LRG1 inflicts profound mitochondrial damage via the ANXA2/AKT axis, precipitating an mtROS burst that ultimately drives NETosis.

### 3.8 Lrg1 Deficiency Enhances Chemo‐Immunotherapy Efficacy via Vascular Normalization

To evaluate the clinical implications of the LRG1‐NETs axis in therapeutic response, we retrospectively analyzed a cohort of 29 BCa patients who received neoadjuvant combination therapy comprising cisplatin and a PD‐1 inhibitor. IHC and IF staining were performed on the matched resected specimens to evaluate the expression of LRG1 and the NET‐specific marker H3Cit (Figure 7A). Patients were subsequently stratified based on their objective pathological response. Baseline clinical characteristics, including age, sex, clinical TNM stage, and initial tumor burden, were well‐balanced between the responder and non‐responder groups with no significant differences (*p* > 0.05; Table S4). Strikingly, compared to the responder group (LRG1‐high: 4/17; NETs‐high: 5/17), the non‐responder group exhibited a synchronous and dramatic upregulation of both LRG1 (9/12) and NETs (10/12) within the tumor microenvironment (Figure 7A–C). These clinical observations strongly implicate the hyperactivation of the LRG1‐NETs axis as a critical physical barrier restricting chemo‐immunotherapy efficacy in BCa.

**FIGURE 7:**
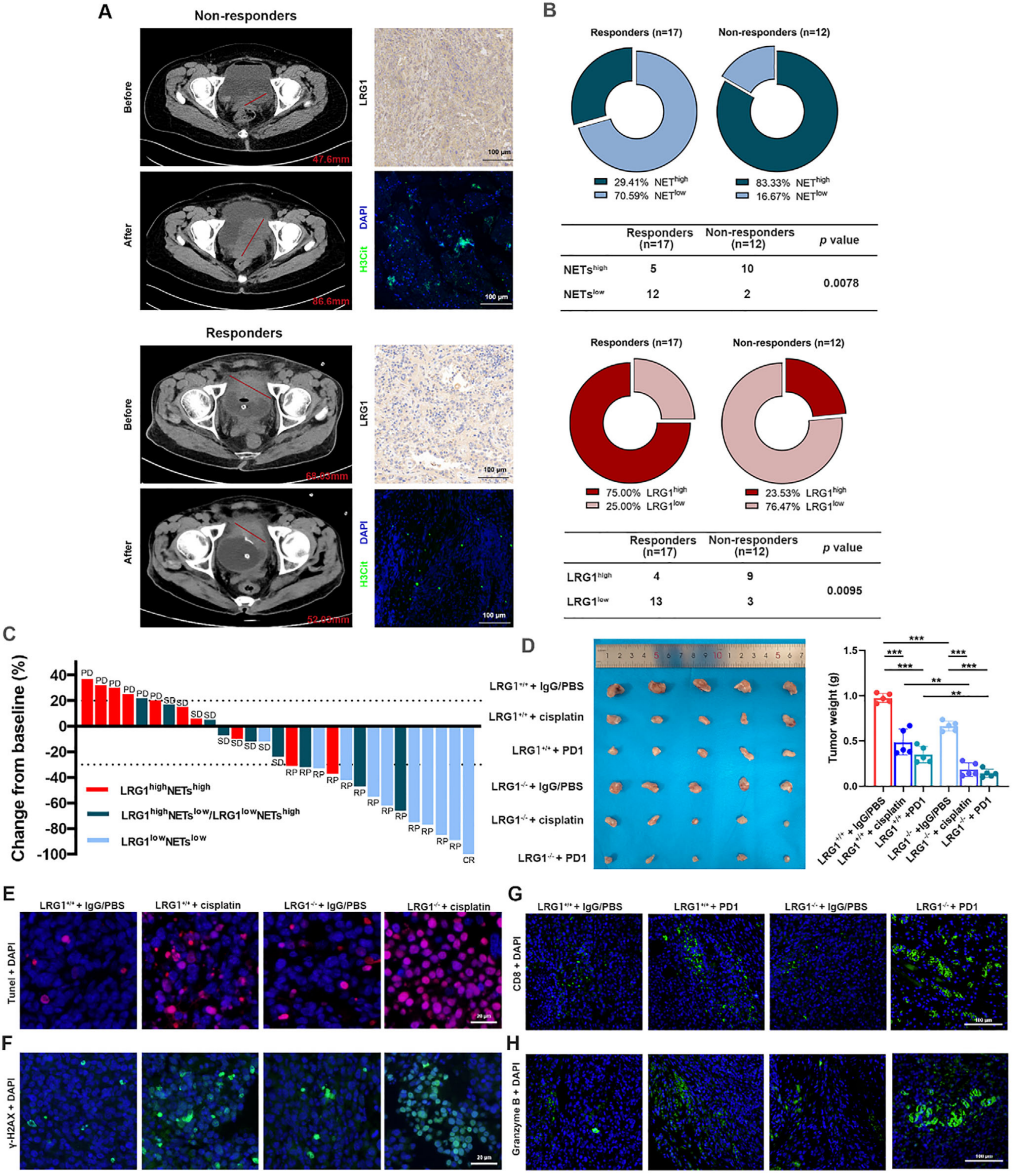
Lrg1 deficiency sensitizes bladder cancer to chemo‐immunotherapy via vascular normalization. (A) Representative pre‐ and post‐treatment computed tomography (CT) scans, alongside matched IHC (LRG1) and IF (H3Cit) images, from responder and non‐responder BCa patients receiving neoadjuvant cisplatin plus PD‐1 blockade. (B) Proportions of LRG1‐high/low and NETs‐high/low patients stratified by objective pathological response (Responders vs. Non‐responders). (C) Bar graph illustrating the percentage change from baseline in the sum of target lesion diameters, evaluated by modified Response Evaluation Criteria in Solid Tumors (mRECIST). (D) Representative macroscopic images and tumor weights of Lrg1+/+ and Lrg1‐/‐ mice treated independently with cisplatin or anti‐PD‐1 antibody. (E, F) Immunofluorescence staining for TUNEL (E) and γ‐H2AX (F) in the cisplatin‐treated cohorts, indicating enhanced drug penetration and cytotoxicity. (G, H) Immunofluorescence analysis of CD8+ T cell infiltration (G) and Granzyme B expression (H) in the anti‐PD‐1 treated cohorts.

Given our preceding finding that Lrg1 deficiency drives tumor vascular normalization, we hypothesized that this structural remodeling could overcome physical barriers within the TME, thereby enhancing the delivery and efficacy of therapeutic agents. To rigorously test this, we subjected Lrg1^−/−^ and Lrg1^+/+^ tumor‐bearing mice to combined cisplatin and anti‐PD‐1 therapy. As anticipated, genetic ablation of Lrg1 profoundly synergized with the combination regimen, yielding superior tumor growth inhibition compared to wild‐type controls (Figure 7D). Mechanistically, to assess chemotherapy enhancement, immunofluorescence analysis was performed. We observed a substantial increase in γ‐H2AX‐positive DNA double‐strand breaks and TUNEL‐indicated apoptosis in the tumors of cisplatin‐treated Lrg1^−/−^ mice. This confirms that the absence of LRG1 structurally improves vascular perfusion, facilitating the deep penetration and intratumoral accumulation of cytotoxic agents. Furthermore, regarding immune modulation, Lrg1^−/−^ mice receiving anti‐PD‐1 therapy displayed a markedly augmented infiltration of CD8^+^ cytotoxic T lymphocytes. Consistent with this enhanced spatial accessibility, these infiltrating CD8^+^ T cells exhibited elevated expression of Granzyme B (Figure 7C,E), denoting a robust reinvigoration of anti‐tumor cytotoxic immunity. Collectively, these data demonstrate that Lrg1 deficiency sensitizes bladder cancer to chemo‐immunotherapy by normalizing the vascular niche and dismantling the immunosuppressive microenvironment.

## 4 Discussion

The clinical management of advanced BCa is severely hindered by the formation of an immunosuppressive microenvironment and dysfunctional tumor vasculature, which collectively build a physical and biological barrier against systemic therapies [20]. While classical anti‐angiogenic strategies predominantly target the VEGF/VEGFR axis, their clinical benefit is frequently undermined by adaptive resistance and exacerbated hypoxia [21]. In this study, we demonstrate that LRG1 is a critical driver of pathological angiogenesis in bladder cancer by disrupting neutrophil mitochondrial homeostasis. We show that tumor‐derived LRG1 binds to ANXA2, triggering an mtROS‐dependent release of NETs that strips mural cells and destabilizes the vasculature. Importantly, this LRG1‐ANXA2‐NETosis axis likely synergizes with the established endothelial TGF‐β/ALK1 pathway. By concurrently driving immature neovascularization and triggering NETosis‐mediated mural cell loss, tumor‐derived LRG1 orchestrates a coordinated multicellular assault that comprehensively explains the profoundly disorganized and leaky vasculature in BCa.

Unlike canonical angiogenic factors essential for physiological homeostasis, LRG1 is uniquely induced under pathological conditions [22]. Our data establish LRG1 not merely as a biomarker for hematogenous metastasis but as an active disruptor of endothelial‐mural cell interactions. Notably, Lrg1 deficiency did not precipitate vascular pruning but rather induced a classic “vascular normalization” phenotype—characterized by enlarged vessel diameters, reduced intraluminal inclusions, and significantly restored pericyte coverage. This structural maturation is critical, as pericytes provide essential paracrine signals to maintain endothelial barrier function [23]. Thus, LRG1 drives pathological angiogenesis by actively uncoupling this vascular stabilizing network. These in vivo observations further underscore that the total microenvironmental pool of LRG1, derived from both host and tumor sources, cooperatively dictates this pathogenic cascade. We posit that host LRG1 deficiency drops the local LRG1 concentration below the critical threshold required to sustain massive neutrophil infiltration and subsequent NETosis, effectively attenuating the pathogenic NETs burden to facilitate the observed structural vascular normalization. Importantly, because LRG1 is primarily a pathological angiogenic factor, its systemic ablation does not perturb physiological vascular homeostasis. Consistent with this, our global LRG1‐/‐ mice exhibited no macroscopic anomalies, and histopathological evaluation of major organs (heart, liver, spleen, lungs, and kidneys) revealed no structural or vascular defects, underscoring the safety and tumor‐specificity of targeting the LRG1 axis.

A paradigm‐shifting discovery of our study is that LRG1‐mediated vascular destabilization relies heavily on the innate immune system. High‐resolution single‐cell transcriptomics and in vivo depletion assays confirmed that LRG1 promotes the profound enrichment of a NET‐forming neutrophil subpopulation within the TME. While the crosstalk between tumor cells and the endothelium has been extensively studied, the intricate tripartite interaction involving innate immune cells remains less defined. Tumor‐associated neutrophils (TANs) exert pleiotropic pro‐tumoral activities [24]. Once activated, they are known to act as pivotal orchestrators across various models of tumor angiogenesis by secreting a diverse repertoire of soluble angiogenic factors—including VEGF‐A, ANGPT1, CXCL8, HGF, and MMP‐9—and by robustly forming NETs [25]. These extruded web‐like structures, composed of decondensed chromatin and highly active granular proteins, not only physically ensnare circulating BCa cells to facilitate metastasis but also exert direct angiopathic effects [26, 27]. Previous studies have indicated that NET‐associated MMP‐9 can degrade the extracellular matrix to release sequestered VEGF, while histone components induce direct endothelial cytotoxicity. Consistent with reports by Aldabbous, Sirois, and colleagues demonstrating that NETs stimulate microvessel sprouting and lumen formation [9, 28], our in vitro permeability assays and in vivo NETs degradation models definitively proved that NETs disrupt endothelial tight junctions and strip pericytes from the vascular wall. This establishes NETs as the missing mechanistic link between LRG1 secretion and vascular collapse.

Mechanistically, how extracellular LRG1 triggers the intracellular execution of NETosis in a sterile tumor microenvironment has been a profound enigma. While recent studies have implicated LRG1 in driving NETosis in diverse contexts—such as diabetic wound healing, corneal fibrosis, and hepatocellular carcinoma—the precise initiating mechanisms within the hypoxic and metabolically rewired TME have remained elusive [29, 30, 31]. Traditionally, NETosis was thought to rely primarily on cytosolic ROS generated by NADPH oxidase (NOX2). However, emerging evidence highlights a paradigm‐shifting mechanism: mtROS‐driven NETosis [32]. Under metabolic stress, mitochondrial electron transport chain dysfunction provokes an mtROS burst, which directly oxidizes mitochondrial DNA (mtDNA) to yield highly immunogenic and pro‐angiogenic NETs [33, 34]. Here, our proteomic and biochemical investigations uncovered a novel signal transduction pathway bridging this gap: the LRR domain of LRG1 directly binds to ANXA2. Phosphorylated ANXA2 acts as a crucial molecular chaperone, shuttling pSer473‐Akt into the mitochondria. Strikingly, LRG1 binding obstructs the phosphorylation of ANXA2 at Tyr24, completely abrogating the mitochondrial translocation of p‐AKT. The resulting loss of mitochondrial p‐AKT signaling precipitates a catastrophic metabolic collapse, culminating in the mtROS burst and subsequent NETs extrusion. Thus, LRG1 acts as a potent “metabolic reprogramming factor” that short‐circuits leukocyte metabolic fidelity, providing a highly innovative perspective on the massive NETs infiltration and vascular abnormalities in bladder cancer.

The ultimate goal of unravelling this pathological axis is to overcome the profound resistance to standard‐of‐care regimens in BCa. Our retrospective clinical cohort analysis validated that the synchronous hyperactivation of LRG1 and NETs constitutes a major hallmark of non‐responders to neoadjuvant cisplatin and PD‐1 blockade. It is well established that the structural immaturity and lack of mural cell coverage in tumor vessels cause profound hypoperfusion and aberrant hemodynamics, which severely restrict the deep intratumoral penetration of chemotherapeutic agents [35, 36]. Furthermore, the extensive web‐like scaffolds of NETs serve as a formidable physical barricade [37]. These extracellular chromatin networks exert strong steric hindrance, directly impeding the transendothelial migration and subsequent stromal infiltration of cytotoxic T cells, thereby perpetuating an immunosuppressive, “cold” tumor microenvironment [38, 39]. By employing Lrg1^−/−^ mice treated independently with either cisplatin or anti‐PD‐1 blockade, we unequivocally demonstrated the dual sensitizing potential of targeting this axis. The structural vascular normalization achieved by Lrg1 ablation effectively dismantled these microenvironmental barriers. For chemotherapy, it restored functional perfusion to facilitate the deep tissue penetration of cisplatin, as evidenced by massive γ‐H2AX accumulation and apoptosis. Parallelly, for immunotherapy, it rescued the spatial exclusion of CD8+ T cells. Ultimately, this vascular remodeling not only amplifies direct chemical cytotoxicity but also reinvigorates Granzyme B‐secreting T lymphocytes, successfully converting a therapy‐refractory tumor into a highly susceptible state.

Despite the robust mechanistic and translational insights provided herein, our study highlights certain limitations that pave the way for future investigations. First, we observed a molecular weight discrepancy between neutrophil‐derived and tumor‐derived LRG1, primarily attributed to differential glycosylation. While neutrophil‐exocytosed LRG1 retains binding affinity for certain targets [40], aberrant glycosylation patterns in various cancers suggest that sugar chain alterations influence LRG1 function in oncogenic contexts [41, 42]. We hypothesize that the specific glycosylation profile of tumor‐derived LRG1 may confer a distinct pathological advantage—potentially enhancing its binding affinity for ANXA2 or improving its stability within the tumor microenvironment. Deciphering the precise structural consequences of this tumor‐specific glycosylation represents a fascinating direction for future research. Furthermore, from a methodological perspective, while our in vivo studies utilized DNase I to effectively dismantle NETs, we acknowledge that this pharmacological approach indiscriminately degrades all extracellular DNA, including cfDNA from necrotic cells. Therefore, future studies employing neutrophil‐specific PAD4 conditional knockout models are warranted to provide more exclusive and definitive genetic evidence for the role of NETs in this LRG1‐driven vascular destabilization. Additionally, while HUVECs were utilized for our in vitro assays to model fundamental vascular responses, we acknowledge that the tumor vasculature exhibits pronounced organ‐specific phenotypes. Future investigations employing bladder‐specific microvascular endothelial cells are warranted to fully validate these findings within the unique organotypic context of the bladder microvascular niche.

## 5 Conclusion

In conclusion, our findings establish LRG1 as a master orchestrator of pathological angiogenesis and immune evasion in bladder cancer via the induction of mtROS‐driven NETosis. Targeting the LRG1‐NETs axis offers a promising therapeutic strategy to induce tumor vascular normalization, reprogram the immunosuppressive microenvironment, and ultimately overcome the current clinical bottlenecks in chemo‐immunotherapy for advanced bladder cancer.

## Author Contributions

**C.D**.: Writing – original draft, Project administration, Funding acquisition, Data curation, Conceptualization. **Z.C**.: Writing – original draft, Visualization, Data curation, Funding acquisition. **X.S**.: Writing – Original draft, Visualization, Investigation. **Z.Y**.: Writing – original draft, Data curation. **C.H**.: Writing – original draft, Visualization. **W.C**.: Writing – original draft, Formal analysis, Data curation. **F.J**.: Writing – original draft, Formal analysis. **Y.L**.: Writing – review & editing, Investigation, Funding acquisition, Conceptualization. **Z.Y**.: Writing – review & editing, Funding acquisition, Formal analysis.

## Funding

This work was supported by the Postdoctoral Innovation Program of Shandong Province (No. SDCX‐ZG‐202502072), the Youth Project of Shandong Provincial Natural Science Foundation (Approval Number: ZR2023QH092, ZR2024QH575 and ZR2026QC0869), the Shandong Provincial Natural Science Foundation General Program (No. ZR2025MS1463), the National Natural Science Foundation of China under the Young Scientists Fund (Approval Number: 82303239), and the Shandong Pingzheng Biotechnology Co., Ltd. (No. 6010123019).

## Ethics Statement

This study was approved by the Ethics Committee of Qilu Hospital of Shandong University (no. KYLL‐202603‐031) and the Animal Ethics Committee of Qilu Hospital of Shandong University (no. DWLL‐202500043). All patients provided written informed consent prior to participation.

## Conflicts of Interest

The authors declare no conflicts of interest.

## Supporting information




**Supporting File**: advs76604‐sup‐0001‐SuppMat.docx.

## Data Availability

The data that support the findings of this study are available from the corresponding author upon reasonable request.
