# Supplementary material for: LRG1 Drives Pathological Angiogenesis by Disrupting Neutrophil Mitochondrial Homeostasis in Bladder Cancer
Source: Adv Sci (Weinh). 2026 Jul 13:e76604. Online ahead of print. doi: 10.1002/advs.76604 (PMC13360113; doi:10.1002/advs.76604)
Supplement: Supplementary file 1 — Supporting File: advs76604‐sup‐0001‐SuppMat.docx. [file ADVS-9999-e76604-s001.docx]

**Supplementary Table S1** Detailed information of antibodies used for WB and IP.

| Name | Cat# | Company name | Dilute concentration | Experiment |
| --- | --- | --- | --- | --- |
| α-Tubulin | GB11200 | Servicebio | 1: 3000 | WB |
| LRG1 | A7850 | Abclonal | 1:1000 | WB |
| ANXA2 | 11256-1-AP | Proteintech | 1:5000 | WB |
| p-AKT (S473) | ET1607-73 | HUABIO | 1:5000 | WB |
| p-ANXA2 (Y24) | sc-135753 | Santa Cruz | 1:500 | WB |
| VDAC1 | ET1601-20 | HUABIO | 1:10000 | WB |
| anti-rabbit IgG, HRP-linked Antibody | AB0101 | Abway | 1:10000 | WB |
| anti-mouse IgG, HRP-linked Antibody | AS003 | Abclonal | 1:2000 | WB |
| ANXA2 | 11256-1-AP | Proteintech | / | IP |
| LRG1 | ab170953 | Abcam | / | IP |
| Control IgG | AC005 | Abclonal | / | IP |

**Supplementary Table S2** Detailed information of antibodies used for immunofluorescence.

| Name | Cat# | Company name | Dilute concentration | Experiment |
| --- | --- | --- | --- | --- |
| LRG1 | 13224-1-AP | Proteintech | 1:200 | IF |
| ANXA2 (mouse) | PTR1321 | Immunoway | 1:100 | IF |
| ANXA2 (rabbit) | 11256-1-AP | Proteintech | 1:200 | IF |
| α-SMA | BM0002 | Boster | 1:500 | IF |
| CD31 | A01513-3 | Boster | 1:100 | IF |
| TOM20 | HA601454 | HUABIO | 1:200 | IF |
| p-AKT (S473) | ET1607-73 | HUABIO | 1:200 | IF |
| LAMP1 | 15665 | CST | 1:50 | IF |
| His-tag | AE086 | Abclonal | 1:50 | IF |
| MPO | YM4746 | Immunoway | 1:100 | IF |
| H3Cit | ab5103 | Abcam | 1:100 | IF |
| CD8A | PT0117R | Immunoway | 1:100 | IF |
| Granzyme B | A2557 | Abclonal | 1:50 | IF |
| Ly6G | YM8307 | Immunoway | 1:100 | IF |
| Laminin | ab11575 | Abcam | 1:200 | IF |
| Anti-Rabbit IgG (AF488) | AB0141 | Abway | 1:100 | IF |
| Anti-Mouse IgG (AF594) | AF594 | Abway | 1:100 | IF |

**Supplementary Table S3.** List of genes in the NET-associated signature used for enrichment analysis.

| H2ab1 | Cr1l | Ncf1 | Plcg2 | H2ac11 | H2al1n |
| --- | --- | --- | --- | --- | --- |
| H4c17 | Ctsg | Ncf2 | H2al1j | H2ac12 | Gm5396 |
| H2al2b | Cyba | Ncf4 | Akt3 | H2ac15 | Macroh2a2 |
| H2al1a | Cybb | Nfkb1 | Tlr2 | H2ac22 | Hdac1 |
| H2al1b | Fcgr1 | Padi4 | Fcgr4 | H2ac24 | Elane |
| H2al1c | Fcgr3 | Pik3ca | H3c7 | H2ac4 | H2az1 |
| H2al1d | Fga | Pik3cd | Map2k1 | H2ac10 | H2al1k |
| H2al1f | Fpr2 | Pik3r1 | Map2k2 | H2ac20 | H2al1e |
| H2al1g | Fpr-rs3 | Pik3r2 | Map3k7 | H2bc1 | H2al2c |
| H2al1h | Fpr-rs4 | Pik3r3 | Mapk1 | H2bc3 | Hdac7 |
| H2al1i | Fpr1 | Prkca | Mapk13 | H2bc6 | Clec7a |
| Gm38574 | Fpr3 | Prkcb | Mapk14 | H2bc7 | Mtor |
| Gm6749 | Gp1ba | Prkcg | Mapk3 | H2bc8 | H2ac21 |
| Ppif | H3c14 | Plcb1 | Macroh2a1 | H2bc9 | H2ab2 |
| Hat1 | H3f3a | Plcb2 | Mapk12 | H2bc11 | H2ab3 |
| Fgb | H3f3b | Plcb3 | H3c3 | H2bc12 | H3f3c |
| Raf1 | Hc | Plcb4 | H3c4 | H2bc13 | Aqp9 |
| Actb | Hdac2 | Plcg1 | H3c2 | H2bc14 | H2ac23 |
| Actg1 | Hdac3 | Mapk11 | H3c6 | H2bc15 | H2bc23 |
| Gm47655 | Hdac5 | Rac1 | H3c10 | H2bc22 | H2bc24 |
| Gm44180 | Hdac6 | Rac2 | H3c11 | H2bc18 | H3f5 |
| Ager | H2ac18 | Rela | H3c13 | H2bc21 | H2bc4 |
| Akt1 | H2ax | Selp | H4c3 | H2ac13 | H2al2a |
| Akt2 | Hmgb1 | Selplg | H4c4 | H2ac19 | Gsdmd |
| Slc25a4 | Itga2b | Src | H4c6 | H4c16 | H2bl1 |
| Slc25a5 | Itgal | Hdac4 | H4c9 | Fpr-rs6 | H4c8 |
| C3 | Itgam | Syk | H4c11 | Fpr-rs7 | H2bw2 |
| C5ar1 | Itgb2 | Tlr4 | H4c12 | H4c1 | Hdac8 |
| Casp1 | Itgb2l | Vdac1 | H4c18 | H4c2 | Slc25a31 |
| Casp4 | Itgb3 | Vdac2 | H2ac25 | H2al1o | Atg7 |
| Clcn3 | Tlr7 | Vdac3 | H2ac1 | H3c1 | Pik3cb |
| Clcn4 | Tlr8 | Vwf | H2ac6 | H2bc26 | H2al1m |
| Clcn5 | Hdac10 | Hdac11 | H2ac7 | H3f4 | H2az2 |
| Camp | Mpo | H2aj | H2ac8 | H2al3 | H2bc27 |
| H4c14 | H3c8 | Fgg | Hdac9 | H3c15 |  |

**Supplementary Table S4.** Baseline clinical characteristics of BCa patients receiving neoadjuvant therapy.

| **Characteristic** | **Total Cohort**  **(n = 29)** | **Responders**  **(n = 17)** | **Non-responders (n = 12)** | **P value** |
| --- | --- | --- | --- | --- |
| **Age (years)** |  |  |  |  |
| Median (Range) | 65 (50–78) | 64 (52–75) | 66 (50–78) | 0.42^a^ |
| **Sex, n (%)** |  |  |  |  |
| Male | 22 (75.9%) | 13 (76.5%) | 9 (75.0%) | 0.73^b^ |
| Female | 7 (24.1%) | 4 (23.5%) | 3 (25.0%) |  |
| **ECOG Performance Status, n (%)** |  |  |  |  |
| 0 | 20 (69.0%) | 12 (70.6%) | 8 (66.7%) | 0.68^b^ |
| 1 | 9 (31.0%) | 5 (29.4%) | 4 (33.3%) |  |
| **Clinical T stage, n (%)** |  |  |  |  |
| cT2 | 15 (51.7%) | 9 (52.9%) | 6 (50.0%) | 0.87^b^ |
| cT3 | 11 (37.9%) | 6 (35.3%) | 5 (41.7%) |  |
| cT4a | 3 (10.3%) | 2 (11.8%) | 1 (8.3%) |  |
| **Clinical N stage, n (%)** |  |  |  |  |
| cN0 | 21 (72.4%) | 12 (70.6%) | 9 (75.0%) | 0.71^b^ |
| cN1–2 | 8 (27.6%) | 5 (29.4%) | 3 (25.0%) |  |
| **Baseline Tumor Burden (cm)** |  |  |  |  |
| Median (Range) | 3.5 (1.5–6.0) | 3.2 (1.5–5.5) | 3.8 (2.0–6.0) | 0.39^a^ |

^a^ Mann-Whitney U test; ^b^ Fisher’s exact test.


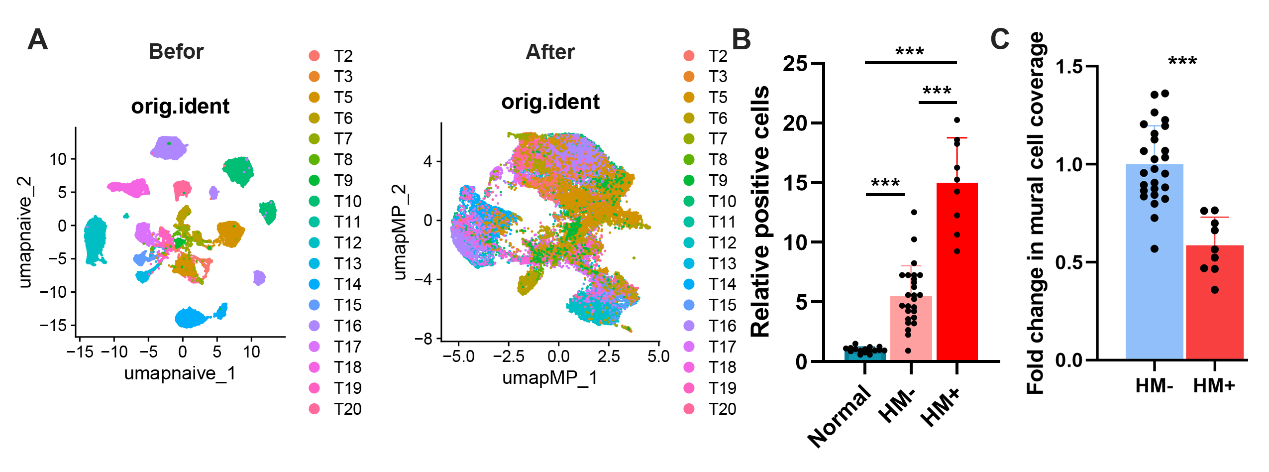


**Supplementary Fig S1.** LRG1 is upregulated in bladder cancer and correlates with hematogenous metastasis and vascular abnormalities. **A.** UMAP visualization of scRNA-seq data before and after Harmony integration. **B.** Statistical quantification of LRG1-positive cells per field. **C.** Quantitative analysis of mural cell coverage.


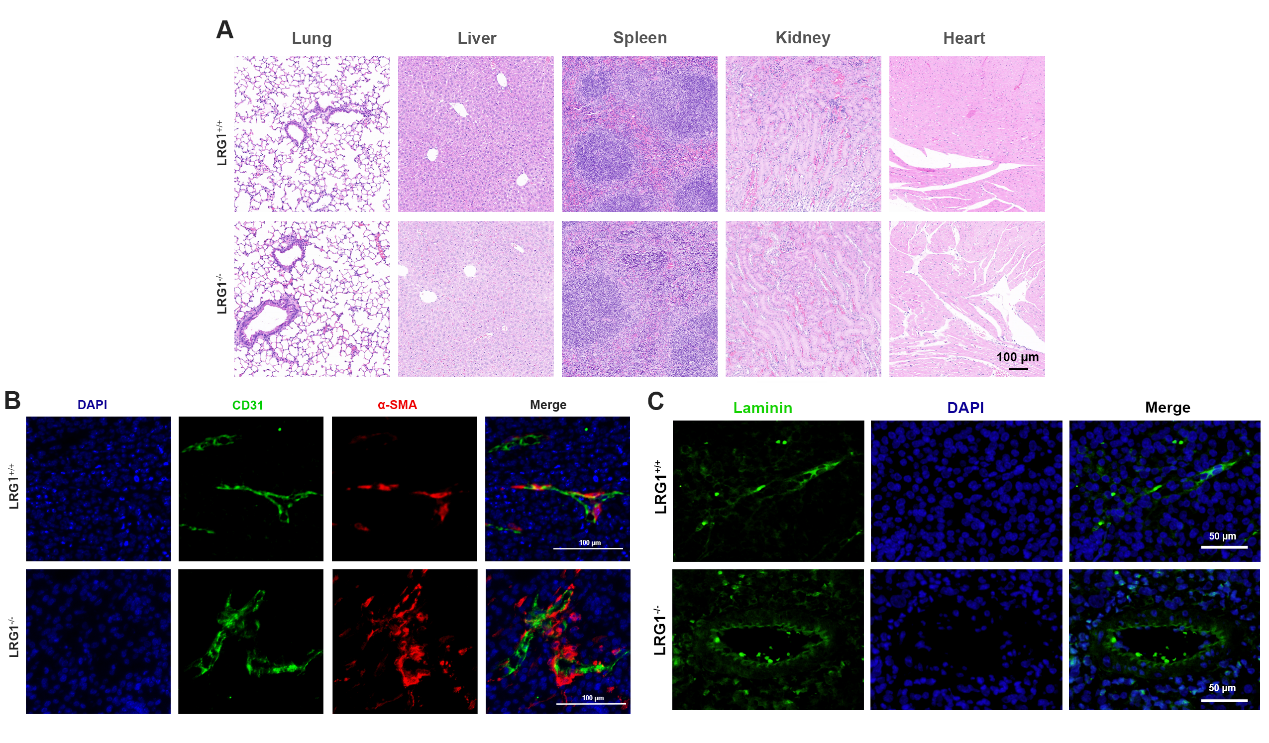


**Supplementary Fig S2.** Systemic safety profile of Lrg1 deficiency and supplementary vascular evaluation. **A.** Representative H&E staining images of major organs obtained from necropsy, demonstrating no observable systemic toxicity or histological abnormalities associated with Lrg1 knockout. **B.** Representative confocal double immunofluorescence images for CD31 (green) and α-SMA (red) evaluating pericyte coverage in the Lrg1^−/−^ orthotopic tumor models. **C.** Immunofluorescence evaluation of basement membrane integrity via Laminin staining.


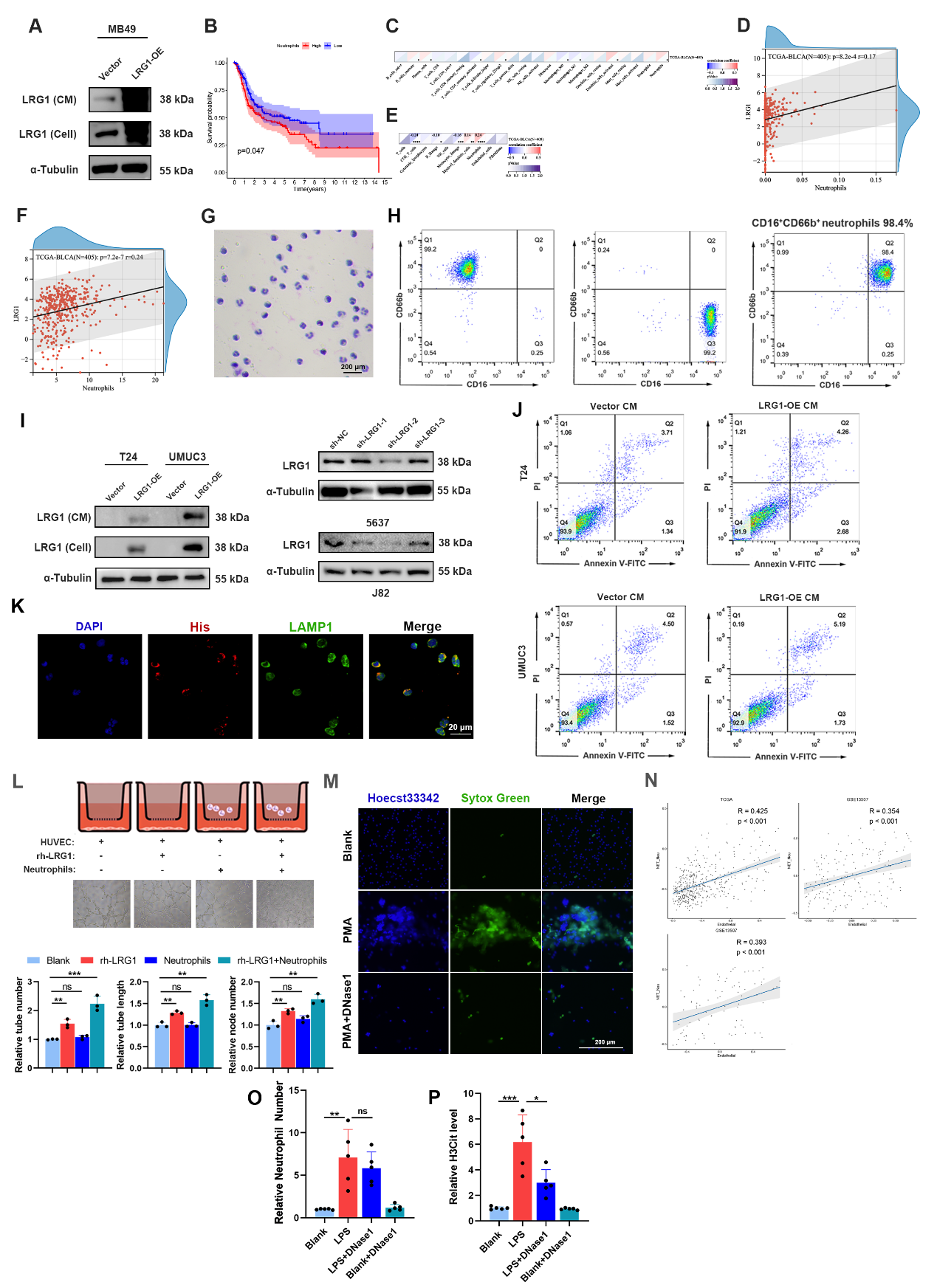


**Supplementary Fig S3.** Validation of primary neutrophil isolation, in vitro functional assays, and NETosis models. **A.** Western blot validation of LRG1 overexpression efficiency in the MB49 Cells. **B.** Survival analysis from pooled datasets indicating that high neutrophil infiltration correlates with poorer survival outcomes. **C-F.** Correlation analysis between LRG1 expression and neutrophil abundance using CIBERSORT (C and E) and MCPcounter (D and F) algorithms based on TCGA data. **G, H.** Verification of isolated primary human neutrophil purity (>95%) utilizing Wright-Giemsa staining (C) and flow cytometric analysis (D). **I.** Western blot verification of LRG1 knockdown (5637/J82) and overexpression (T24/UMUC3) efficiency in BCa cells. **J.** Flow cytometric assessment confirming that LRG1-OE conditioned medium (CM) does not significantly affect the baseline apoptotic rate of neutrophils. **K.** Representative confocal immunofluorescence images showing the colocalization of the recombinant protein tag (His-tag) with the lysosomal marker LAMP1 in neutrophils following rh-LRG1 treatment. **L.** In vitro co-culture assays demonstrating the synergistic amplification of rhLRG1-induced pro-angiogenic effects on endothelial cells in the presence of neutrophils. **M.** Representative SYTOX Green fluorescence images visually confirming the massive release of extracellular DNA webs following PMA stimulation and their efficient dismantling upon DNase I treatment. **N.** GSVA correlation analysis demonstrating a significant positive association between the infiltration abundance of the NCF2_neu subpopulation and endothelial cells in bulk RNA-seq cohorts. **O.** Statistical quantification of infiltrated neutrophils. **P.** Quantitative analysis of H3Cit fluorescence intensity.


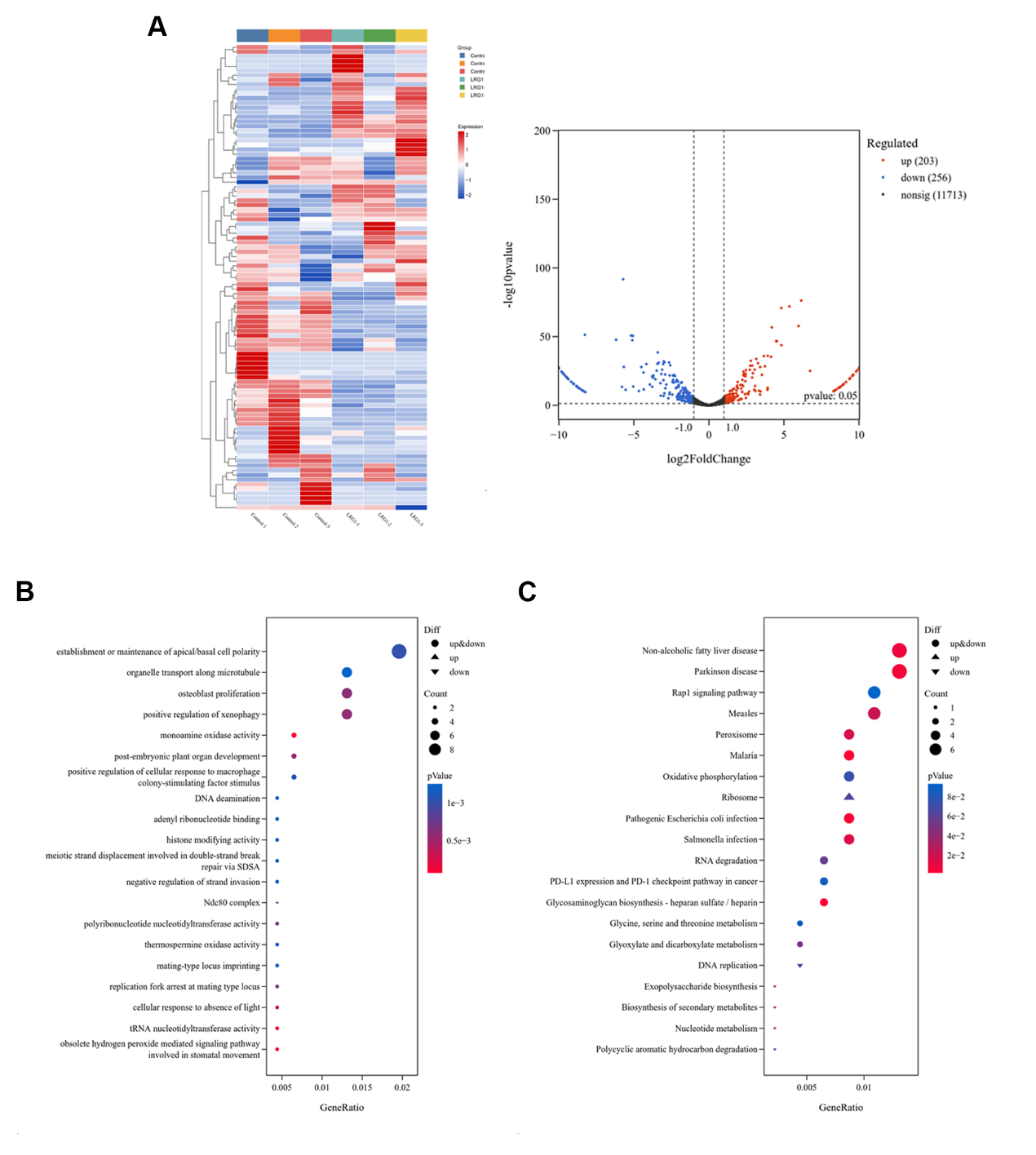


**Supplementary Fig S4.** Transcriptomic profiling reveals metabolic and immunomodulatory reprogramming induced by LRG1 in neutrophils. **A.** Heatmap and volcano plot illustrating the differentially expressed genes in primary neutrophils following LRG1 stimulation. **B.** Gene Ontology (GO) enrichment analysis. **C.** KEGG pathway enrichment analysis.


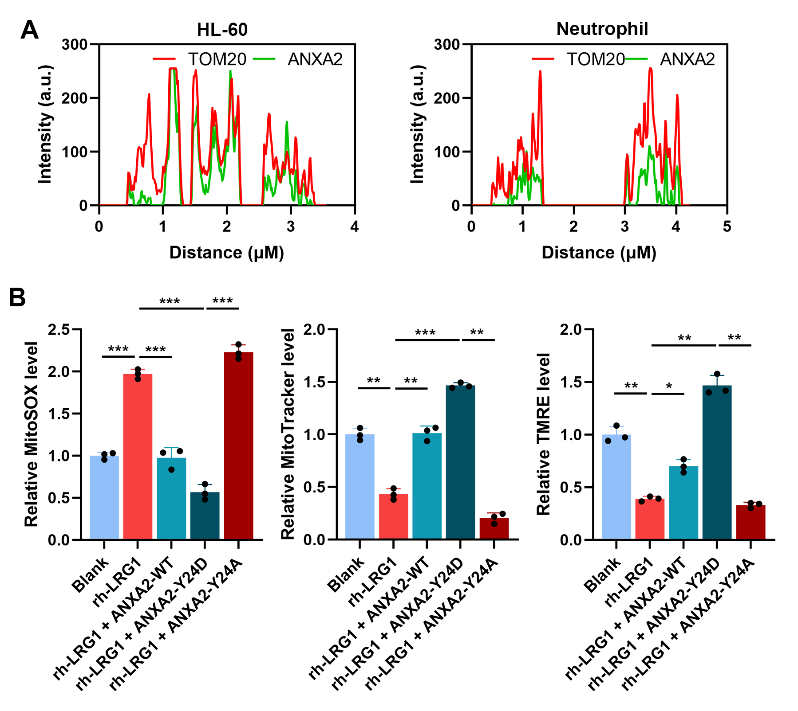


**Supplementary Fig S5.** Mitochondrial localization of ANXA2 and its regulation of mitochondrial homeostasis in neutrophils. **A.** Immunofluorescence colocalization of TOM20 and ANXA2. **B.** Quantitative statistical analysis of mitochondrial homeostasis in neutrophils.
